# Antibiotic Selection for Sustained Local Therapy May Be Important to Reduce *Pseudomonas aeruginosa* Dominance Against *Staphylococcus aureus* and Bone Response Modulation in an Ovine Polymicrobial Fracture-Related Infection Model

**DOI:** 10.3390/microorganisms14092062

**Published:** 2026-09-16

**Authors:** Dustin Williams, Robert Falconer, David Rothberg, Mario Lopez, Jacob Adams, Alice Miller, Richard Tyler Epperson, Brooke Kawaguchi, Caroline Garrett, Paul Pasquina, Nicholas Ashton

**Affiliations:** 1 Department of Orthopaedics, University of Utah, Salt Lake City, UT 84108, USAn.ashton@utah.edu (N.A.); 2 Department of Biomedical Engineering, University of Utah, Salt Lake City, UT 84112, USA; 3 Department of Pathology, University of Utah, Salt Lake City, UT 84112, USA; 4 Department of Physical Medicine and Rehabilitation, Uniformed Services University, Bethesda, MD 20814, USA; 5 Purgo Scientific, Salt Lake City, UT 84112, USA; 6 Noorda College of Osteopathic Medicine, Provo, UT 84606, USA; 7 Office of Comparative Medicine, University of Utah, Salt Lake City, UT 84112, USA; 8 Department of Ophthalmology and Visual Sciences, University of Utah, Salt Lake City, UT 84112, USA; 9 The Center for Rehabilitation Sciences Research, Uniformed Services University, Bethesda, MD 20814, USA

**Keywords:** biofilm, bacteria, fracture-related infection, local delivery, *Pseudomonas aeruginosa*, *Staphylococcus aureus*

## Abstract

Polymicrobial fracture-related infections (FRIs) underpinned by bacterial biofilms pose therapeutic challenges distinct from those of monomicrobial infection, as interspecies dynamics may alter both antimicrobial response and host tissue injury. This study evaluated systemic and local antibiotic strategies in an ovine FRI model co-inoculated with a polymicrobial *Staphylococcus aureus* and *Pseudomonas aeruginosa* biofilm. At 21 days, untreated positive controls retained substantial total bioburden yet paradoxically preserved bone volume and exhibited low osteoclast activity, contrasting with the marked osteolysis previously observed in monomicrobial infection controls using this model. Although untreated controls remained *S. aureus* predominant, their *S. aureus*:*P. aeruginosa* ratio declined from approximately 200:1 at inoculation to 15:1, indicating a progressive ecological shift over time. A systemic levofloxacin plus rifampin regimen more effectively reduced *S. aureus* than *P. aeruginosa*. Likewise, all antibiotic-treated groups shifted from an *S. aureus*-dominant inoculum toward *P. aeruginosa* predominance, consistent with unequal antimicrobial suppression altering polymicrobial pathogen balance. Single-dose local antibiotic powder and calcium sulfate bead strategies provided inconsistent microbiological benefit and failed to preserve bone. Reloadable local antibiotic delivery reduced *S. aureus* tissue bioburden and preserved the greatest bone volume among antibiotic-treated groups but the antibiotics selected for delivery did not prevent persistent *P. aeruginosa* colonization or the shift in pathogen balance. These findings indicate that microbiological burden reduction, residual pathogen composition, and bone preservation may result in distinct treatment outcomes in polymicrobial FRI. This study highlights the need for appropriate antibiotic selection with ecologically informed, dual targeted local and systemic strategies to manage polymicrobial, biofilm-complicated FRIs.

## 1. Introduction

Fracture-related infections (FRIs) remain a persistent and debilitating challenge despite notable advances in pharmacological and technological interventions over the last five decades. FRI complicates approximately 1–2% of closed fractures managed with internal fixation and 30–50% of open fractures; rates correlate with increasing severity [1,2,3,4]. Although systemic antibiotic therapy remains a cornerstone of FRI management, its efficacy against biofilm-associated infection is often limited [5]. Compromised local vasculature impedes drug delivery, and systemic dilution prevents antibiotic concentrations at the fracture site from reaching levels sufficient to penetrate and effectively kill established biofilm [6].

To overcome these limitations, local antibiotic delivery strategies have been developed to achieve therapeutic concentrations directly at the infection site while minimizing systemic toxicity [7]. Common approaches include the topical application of antibiotic powders and the incorporation of antibiotics into carriers such as calcium sulfate (CaSO_4_) beads or polymethylmethacrylate (PMMA) [7,8,9,10]. These bolus delivery methods generate an initially high local antibiotic field; however, they lack sustained release. Effective bactericidal levels typically persist for only 24–72 h with antibiotic powders and decrease exponentially from the carrier surface in bead-based systems [11,12,13,14]. PMMA beads release only a small fraction of their antibiotic payload, and in some cases, less than 5% [15,16]. Although these techniques have shown promise in prophylactic contexts, particularly after high-risk open fractures, their treatment advantages over systemic therapy alone are frequently modest or clinically nonsignificant in established infections [8,9,10,17,18]. The transient exposure characteristic of bolus delivery represents a key limitation: antibiotic levels quickly fall below the minimum biofilm eradication concentration, allowing residual biofilm on hardware and/or local tissues to reseed infection [19,20].

Reloadable local delivery systems, such as the Purgo Pouch, offer a potential solution to these challenges [21]. The Purgo Pouch is a drug-delivery device featuring a refillable reservoir and a percutaneous catheter, allowing sustained, high intrawound antibiotic concentrations throughout treatment. In prior studies using an established ovine long-bone FRI model inoculated with methicillin-resistant *Staphylococcus aureus* (MRSA) biofilms, the Purgo Pouch demonstrated greater efficacy compared to intravenous systemic therapy and antibiotic-loaded CaSO_4_ beads [21]. Subsequent work in this ovine model evaluated systemic therapy against monomicrobial *S. aureus* and *Pseudomonas aeruginosa* biofilms and demonstrated the limitations of systemic therapy alone for biofilm control [5]. Together, these findings support reloadable local delivery as a potential strategy for biofilm-complicated monomicrobial FRI. However, polymicrobial FRIs involve interspecies interactions that alter antimicrobial response and treatment outcomes, necessitating evaluation of reloadable local antibiotic delivery under mixed-species conditions.

Polymicrobial FRIs, involving two or more bacterial species, account for nearly one-third of all clinical cases, with some reports citing incidences as high as 45% [1,22,23]. These cases exhibit greater treatment recalcitrance and are associated with higher surgical debridement rates, greater antibiotic requirements, and poorer clinical outcomes, including elevated risks of amputation, compared to monomicrobial infections [24,25]. *S. aureus* and *P. aeruginosa* are among the most frequently isolated and clinically consequential pathogens in FRI and chronic wound infection, and their complementary resistance profiles complicate combined treatment [23,26,27,28]. Despite their prevalence and clinical importance, in vivo preclinical models incorporating polymicrobial biofilm inoculation of bone or orthopedic implants, alongside systematic evaluations of treatment strategies, remain scarce, particularly those that assess both microbiological control and host tissue outcomes. This gap limits understanding of polymicrobial infection progression, interspecies dynamics, and the extent to which treatment-associated reductions in bacterial burden alter the histological impacts of infection.

To address this limitation, the present study investigated the potential therapeutic efficacy of the Purgo Pouch compared to clinically relevant systemic and local antibiotic treatments, including antibiotic powders and antibiotic-loaded CaSO_4_ beads, in an ovine long-bone FRI model inoculated with polymicrobial *S. aureus* and *P. aeruginosa* biofilms. Microbiological and histological analyses were performed to assess each treatment’s effect on bacterial burden, interspecies interactions, and bone response.

## 2. Materials and Methods

### 2.1. Biofilm Growth

*S. aureus* ATCC 6538 and *P. aeruginosa* ATCC 27853 biofilms were grown on simulated titanium fracture fixation plates in a modified CDC biofilm reactor. The reactor was prepared and autoclaved with eight simulated fracture fixation plates suspended in holding arms, then run using a previously described modified protocol from ASTM standard E2562-07 [21,29]. Briefly, the reactor was aseptically filled with 500 mL of brain heart infusion (BHI) broth (Becton Dickinson and Company, Sparks, MD, USA) and inoculated with diluted 500 µL McFarland suspensions of *S. aureus* and *P. aeruginosa* across five time points using a previously published method [29]. The reactor was placed on a hot/stir plate set at 34.1 °C and 130 rpm. After growing for 24 h in batch culture (no nutrient exchange), 10% diluted BHI was flowed through the reactor at a rate of 6.94 mL/min using a peristaltic pump for an additional 24 h. At 48 h, three plates were immediately quantified as controls to establish baseline bioburden, and the remaining five were transported to the operating room.

### 2.2. Surgical Preparation and Method

Thirty-eight male and female Rambouillet sheep aged 1–3 years and weighing ~50–70 kg were acquired from Robinson Elk Ranch (Richmond, UT, USA) and acclimated for at least 72 h before undergoing any procedures. All procedures were performed with approval and oversight of the Institutional Animal Care and Use Committee (IACUC) at the University of Utah and the United States Army Medical Research and Development Command’s Animal Care and Use Review Office (ACURO) and conducted in accordance with the Animal Welfare Regulations [30] and the Guide for the Care and Use of Laboratory Animals [31]. All work was performed in a fully AAALAC-accredited facility.

Surgical preparation and procedures were performed as previously described [5,21]. Briefly, with sheep under a stable plane of surgical anesthesia with multimodal systemic analgesia, the right hind limb was exposed to a blast (90 psi) 4 in. above the proximal medial aspect of the right hind tibia from an air impact device to simulate soft tissue trauma [32]. Sheep were then transferred to the operating table, and the region from the right mid-tibia to the hip was aseptically prepared for surgery. Sterile Coban was wrapped from the hoof to the distal tibia, and the entire site was surgically draped.

A 5 cm incision was made on the anterior region of the proximal medial aspect of the right tibia, beginning near the tibial tuberosity and extending ~5 cm further distal. The skin and subcutaneous tissue were dissected from the bone surface, the periosteum stripped, and two simulated fractures were created (each ~1 mm wide and 2 cm long) on the bone surface using a custom bone saw. Simulated fracture fixation plates with *S. aureus* and *P. aeruginosa* biofilms grown on them were secured to the bone using cortical bone screws (Movora, St. Augustine, FL, USA). Immediately after, antibiotic powders, antibiotic-loaded beads, or a Purgo Pouch were added to the surgical site (Table 1). The site was sutured closed, bandaged, and the animal recovered.

### 2.3. Local Therapy Preparation

The Purgo Pouch was assembled as described previously [21]. Sterilization was performed using an overnight ethylene oxide cycle, which was previously shown not to affect release kinetics [21]. STIMULAN^®^ CaSO_4_ beads were prepared according to the manufacturer’s instructions and based on trauma surgeon input at the time of study design. On a day of surgery, in a biosafety cabinet, a sterile-gloved researcher combined 240 mg tobramycin, 1 g vancomycin, and the provided CaSO_4_ powder with sterile water, adding an additional 1 mL to facilitate mixing. The resulting paste was molded into 3 mm wells, allowed to cure for 15 min, and transported to the operating room. Antibiotic powder quantities were based on one vial of each drug as supplied at our institution to reflect typical clinical preparation and administration. The tobramycin dose in each Pouch was selected to match the amount loaded into the beads, and the vancomycin dose was reduced from the amount in the beads to mitigate the risk of local inflammation observed in a previous investigation [21].

### 2.4. Animal Treatment Groups

Sheep were randomly assigned to one of five treatment groups (Table 1). All sheep received the same surgery up to the point of local treatment application. Blinding of investigators to treatment group assignment was not performed during animal experimentation or data collection. For Group 5 sheep, pouches were removed after 10 days of daily refilling/treatment as previously described: the residual antibiotic solution was collected, the pouch was filled with 10 mL of 2% lidocaine HCL solution (MWI Animal Health, Chicago, IL, USA) for approximately 1 h, the lidocaine and connectors were removed, the suture anchors were cut away, and the device was pulled out through the percutaneous exit site [21]. A sample size of n = 8 animals per treatment group was determined by a priori power analysis (two-sided Fisher’s exact test, α = 0.05), assuming infection rates of 100% in controls and 0% in treated animals.

### 2.5. Blood Collection and Serum Antibiotic Quantification

Six mL of blood was collected via the jugular vein from each sheep at baseline (time = 0) prior to surgery. Blood was also collected from Group 3 and 4 sheep at 4, 24, 216 h timepoints and immediately prior to euthanasia (504 h). Blood was drawn from Group 5 sheep at 1, 2, 4, 8, 24, 56, 72, 104, 120, 200, 216, 240, and 264 h time points. Samples were placed in Vacutainer tubes, centrifuged at 2500 rpm for 10 min, and the serum was aseptically transferred to sterile cryogenic vials stored at −80 °C until submission to ARUP Laboratories (Salt Lake City, UT, USA) for immunoassay quantification.

### 2.6. Bone Labeling

Animals were intravenously injected with a calcein green (Sigma Aldrich, St. Louis, MO, USA) solution (9.9 mg/kg) via the jugular or cephalic vein on 5 and 16 days before euthanasia, following previously published protocols to facilitate analysis of bone viability and remodeling dynamics [33,34].

### 2.7. Euthanasia and Sample Collection

All sheep were euthanized at 21 days with an intravenous injection of veterinary medical-grade pentobarbital-based euthanasia solution. The right hind limb was immediately detached at the coxofemoral joint, and the incision site was prepped with betadine for microbiological analysis. The original incision was reopened using a sterile scalpel. The soft tissue directly above the distal fixation plate was collected and placed into 40 mL saline. Next, the screws of the distal fixation plate were removed, along with the plate. A section of bone directly under the fixation plate was excised using a trephine, and a second trephine was used to excise the area surrounding the individual screw tracks. Tissue samples were weighed and homogenized. All samples were vortexed in conical tubes for 1 min, sonicated at 40 kHz for 10 min, and then vortexed for an additional 1 min. A 100 µL aliquot of the resulting suspension was taken from each tube and promptly spread onto tryptic soy agar (TSA) as a contamination check. Bacterial counts were subsequently determined using a series of 10-fold serial dilutions. A total of 50 µL of each dilution from each sample was plated on both Baird-Parker Agar and Cetrimide Agar and dispersed in 5 × 10 µL spots to selectively isolate *S. aureus* and *P. aeruginosa*, respectively. Dilutions were prepared and plated sequentially through the seventh 10-fold dilution. Plates were incubated at 37 °C for 24–48 h to allow bacterial growth. Colony counts were used to represent bioburden as CFU/sample for hardware components and were normalized to CFU/gram for tissues.

### 2.8. Histological Processing

At necropsy, the proximal fixation plate and adjacent soft tissue were isolated with an oscillating bone saw. The sample was then fixed in 10% neutral buffered formalin and retained in 70% ethanol until further processing. Three-dimensional reconstructions of the bone surface directly under the proximal fracture fixation plate were created utilizing µ-CT scans to measure the amount of bone retained following treatment [21]. The samples were further dehydrated in increasing concentrations of ethanol (70%, 80%, 95%, 100%) and xylene and embedded in PMMA. The embedded samples were cut, sectioned, and polished to an optical finish. Backscattered electron (BSE) imaging was used to analyze the percentage of woven bone growth stemming from the surgical site under the plate. The sections were then further ground to ~75 microns for fluorescence microscopy to visualize the calcein green label to confirm bone viability and growth. Sections were finally stained with Sanderson’s rapid bone stain to qualitatively image sections and enumerate the osteoclast count. For osteoclast quantification, two standardized 20× fields (1.11 × 0.74 mm; 0.817 mm^2^) were evaluated per animal, one on each side of the simulated fracture, excluding the osteotomy/fracture region and plate edges. Osteoclasts were defined as multinucleated cells on or near bone surfaces within resorption areas on SRBS-stained sections and were counted manually by a single blinded observer. Counts were normalized to total region of interest (ROI) area and reported as osteoclasts/mm^2^.

### 2.9. Statistical Methods

Statistical processing and analysis were conducted using Stata Statistical Software: Release 18 (StataCorp LLC., College Station, TX, USA). Microbiological samples that recovered no bacteria were reported at the limit of detection by artificially adding one colony to the least diluted plate. Bacterial reductions in hardware and residual tissue bioburdens were summarized by treatment-group means and compared using two-sample *t*-tests with unequal variances (Welch *t*-tests). Comparisons of woven bone percentage measured by scanning electron microscopy were similarly evaluated using Welch *t*-tests.

Osteoclast counts and bone volume (µ-CT) were analyzed using mixed-effects linear regression models to account for multiple measurement locations within each animal, with experimental group specified as a fixed effect and individual sheep as a random effect. A *p*-value < 0.05 was considered statistically significant. To account for multiple testing across the family of pairwise comparisons, a false discovery rate control procedure (Benjamini–Krieger–Yekutieli method) was applied separately to the family of pairwise comparisons for each endpoint, and statistical significance was determined based on these adjusted *p*-values. Formal normality testing was not performed due to the small sample size, which limits the reliability of such tests.

Investigators were not blinded to treatment assignment during surgery because the treatment materials and their methods of application were visibly distinguishable. Microbiological samples were collected by the surgical team at the animal facility, and these investigators were therefore aware of treatment assignment during sample collection. Histological imaging and data collection were performed under blinded conditions. Each animal was identified only by a unique number assigned by the animal facility, and treatment assignments were not available to the bone pathologist during image acquisition or qualitative histological assessment, woven bone measurements, or osteoclast counting. Treatment assignments were revealed only after all specimen-level imaging and measurements were completed for group organization and statistical analysis.

## 3. Results

### 3.1. Biofilm Growth

**Figure 1:**
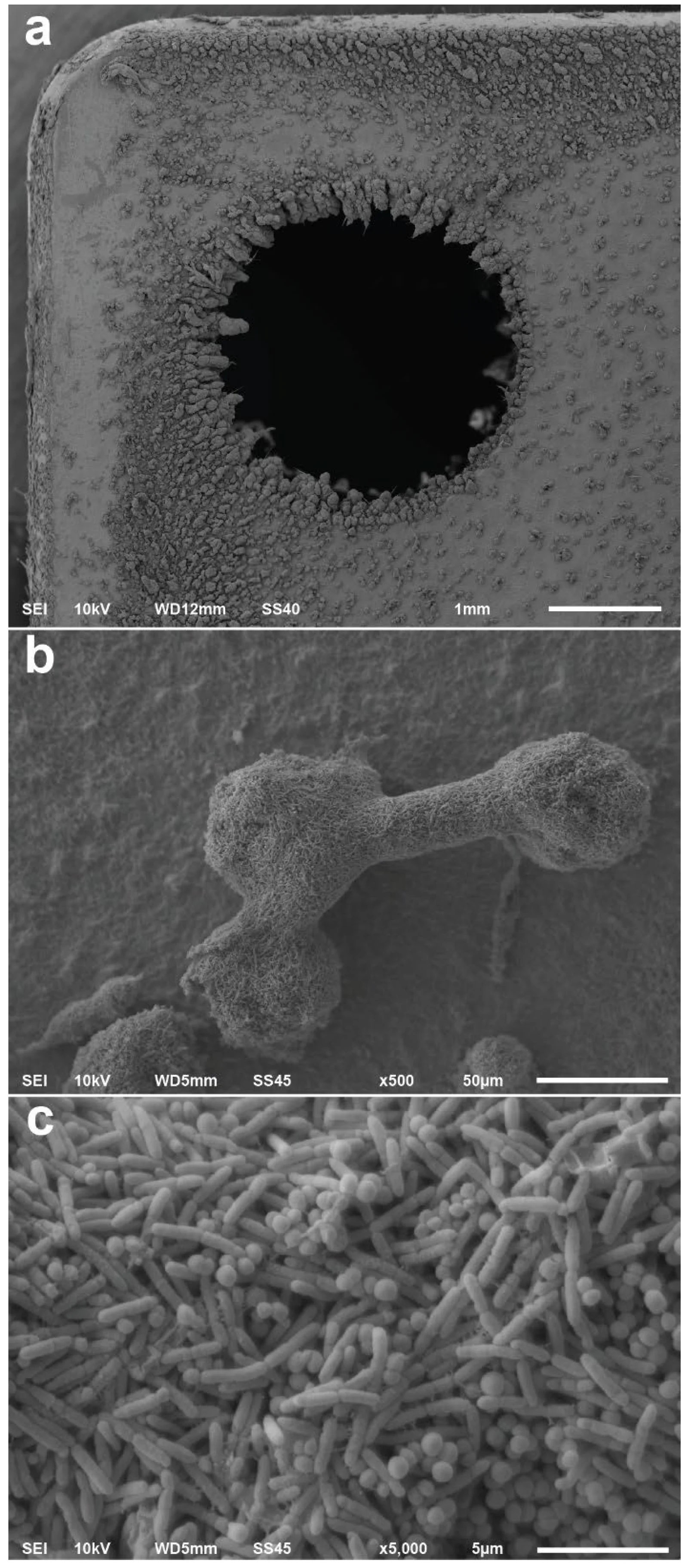
Representative images of *S. aureus* and *P. aeruginosa* polymicrobial biofilms grown on simulated fracture fixation plates made of titanium. (**a**) Stitched SEM micrographs showing biofilm growth on the top corner of the simulated fracture fixation plate. (**b**) Polymicrobial biofilm morphology displaying three-dimensional, cotton ball-like structures. (**c**) Higher resolution image displaying the individual *S. aureus* (cocci) and *P. aeruginosa* (bacilli) cells. Adapted from Kay et al. [29].

Each biofilm-ridden simulated fracture fixation plate contained 9.41 ± 0.59 log_10_ CFUs of *S. aureus* and 7.32 ± 0.89 log_10_ CFUs of *P. aeruginosa* (mean ± standard deviation). Animals were inoculated with a bacterial ratio of ~200:1 (*S. aureus*:*P. aeruginosa*). Quantification of the representative control plates established that all animals had mature biofilms successfully inoculated at surgery. Representative biofilms are shown in Figure 1.

### 3.2. Animal Model Outcomes and Infection Observations

Daily check sheets indicated that animal pain, distress, and lameness were well managed with perioperative analgesia and supportive care. All animals developed an infection, as evidenced by observable heat, pain, redness, and swelling. One animal in Group 5 died prematurely on post-op day 4 due to unrelated respiratory conditions that were determined to have originated from preexisting subclinical illness. A contingent animal was approved in the protocol and used to complete the study. All other animals survived to the 21-day endpoint.

### 3.3. Antibiotic Blood Serum Quantification

All locally applied antibiotics resulted in undetectable levels in most serum samples. Specifically, all samples from animals receiving local powders (Group 3) were below the limit of detection (LOD) for tobramycin (0.4 µg/mL) and vancomycin (1.1 µg/mL). All samples from animals receiving antibiotic-loaded CaSO_4_ beads (Group 4) were below the LOD, except for two vancomycin samples at 24 h (1.4 and 1.5 µg/mL). Blood serum concentrations also remained below the LOD for pouch-treated animals (Group 5), except for three vancomycin samples (1.3 µg/mL at 56 h, 1.3 µg/mL at 200 h, and 1.4 µg/mL at 216 h).

### 3.4. Microbiological Outcomes

Supplemental statistical analyses of individual sample bioburdens, figures, and effect size calculations are available in Supplementary Materials.

#### 3.4.1. Hardware Reduction

**Figure 2:**
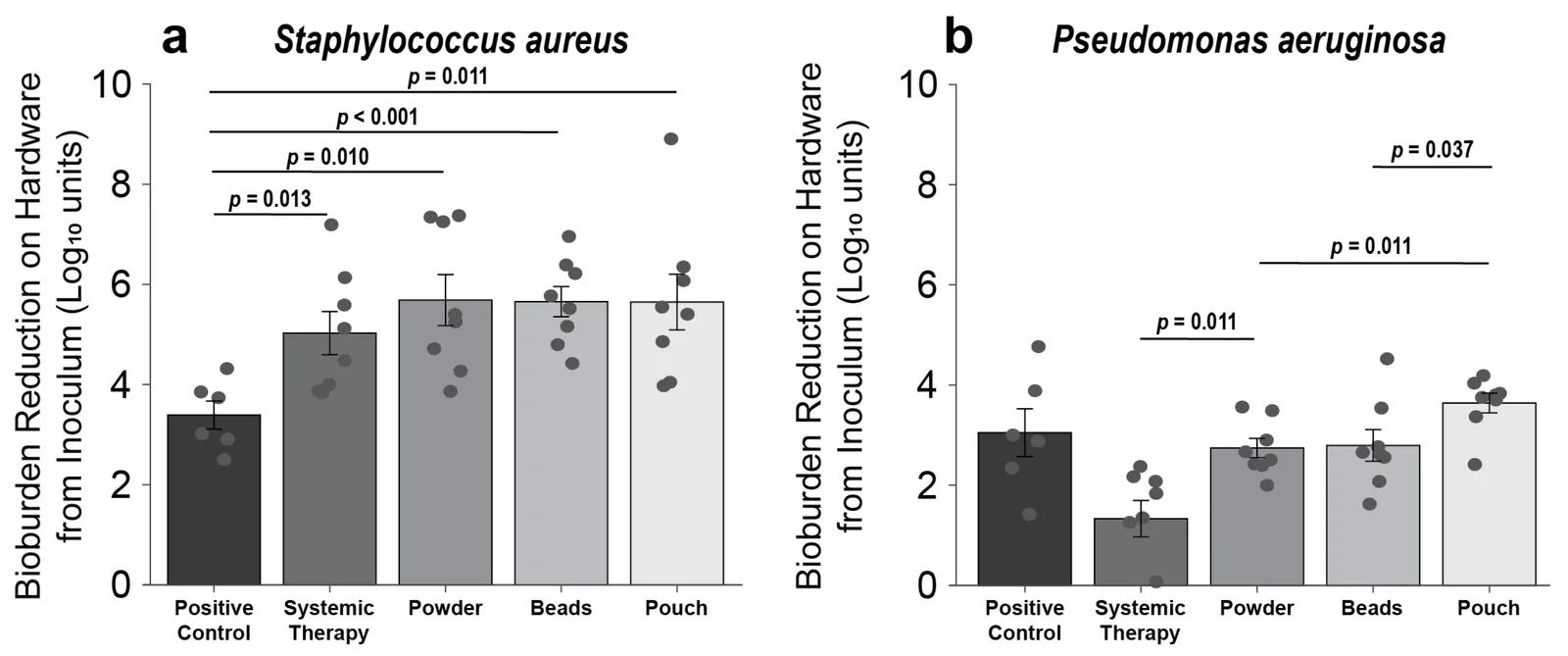
Bioburden reductions in orthopedic hardware (fixation plates + screws). The average hardware bioburden reduction, measured by the difference between the bioburden at inoculation and during necropsy, is reported for (**a**) *S. aureus* and (**b**) *P. aeruginosa*, in log_10_ units. Axes are shown from 0 to 10 for both bacteria to permit direct visual comparison. One observation for the systemic therapy group in subplot B had a value of −0.47 and is not on the graph, but it was used in all analyses. Group means were compared using two-sample *t*-tests with unequal variances (Welch *t*-tests), with *p*-values adjusted for multiple comparisons using a false discovery rate procedure (Benjamini–Krieger–Yekutieli). Statistical comparisons are reported in Table 2. Error bars represent ± standard error. Additional individual sample analyses in Supplementary Materials.

Bioburden reduction in fixation hardware (fixation plates and screws) followed distinct patterns for *S. aureus* and *P. aeruginosa* (Figure 2). For both organisms, locally delivered therapies achieved greater reductions than systemic therapy alone (Figure 2).

Systemic therapy significantly reduced *S. aureus* bioburden compared with the positive control (*p* = 0.013; Table 2). While animals receiving adjunctive local treatments (powders, beads, or the pouch) showed further reductions over systemic therapy alone, these comparisons were not statistically significant. No significant differences were detected among the local therapy groups, whose mean bioburdens varied by less than 0.1 log_10_ units.

*P. aeruginosa*-inoculated animals receiving systemic therapy alone exhibited a lesser reduction in hardware bioburden from the inoculum than positive controls (*p* = 0.018; Table 2). All local therapies resulted in significantly greater reductions than systemic therapy alone, although reductions in the powder- and bead-treated groups remained lower than those in the positive control. Pouch therapy achieved the greatest reduction in *P. aeruginosa* bioburden, which was statistically significant compared with the powder and bead groups (*p* = 0.011 and *p* = 0.037, respectively; Table 2), but not from the positive control (*p* = 0.219; Table 2).

#### 3.4.2. Remaining Tissue Bioburden

**Figure 3:**
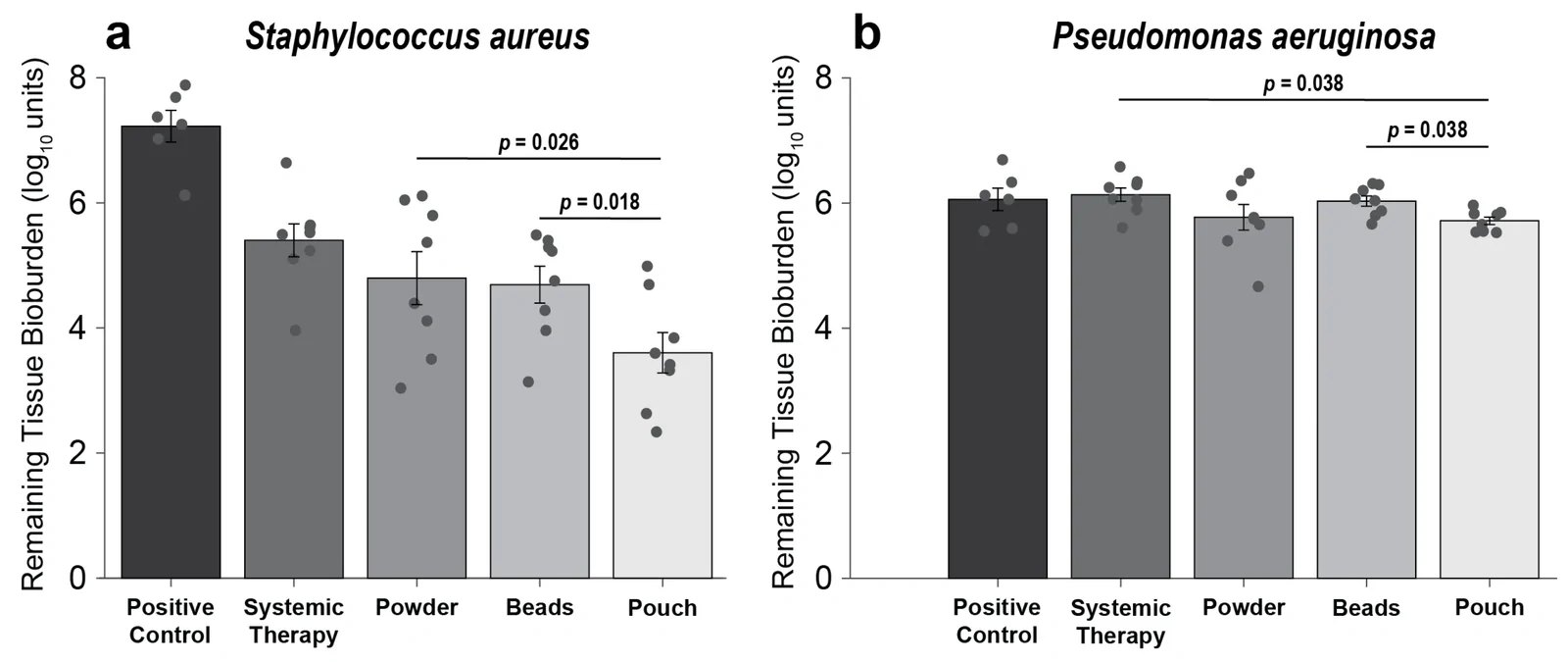
Bioburden levels recovered in tissue samples (soft tissue + bone). The average tissue bioburden, measured by the total bacteria recovered during necropsy, is reported for (**a**) *S. aureus* and (**b**) *P. aeruginosa*, in log_10_ units. Group means were compared using two-sample *t*-tests with unequal variances (Welch *t*-tests), with *p*-values adjusted for multiple comparisons using a false discovery rate procedure (Benjamini–Krieger–Yekutieli). Statistical comparisons are reported in Table 2. Error bars represent ± standard error. Additional individual sample analyses in Supplementary Materials.

The remaining tissue bioburden after treatment diverged between *S. aureus* and *P. aeruginosa* (Figure 3). Antibiotic therapies markedly killed *S. aureus* relative to the positive control but had minimal effect on *P. aeruginosa*. Systemic therapy significantly decreased *S. aureus* bioburden compared to the positive control (*p* < 0.001; Table 2). All local therapies further reduced *S. aureus* recovery versus systemic therapy, although only the pouch treatment achieved statistical significance (*p* = 0.001; Table 2). Tissue bioburden in pouch-treated animals was also significantly lower than in powder- and bead-treated groups (*p* = 0.026 and *p* = 0.018, respectively; Table 2).

By contrast, *P. aeruginosa* bioburden remained similar across all treatment groups, including positive controls. Animals receiving systemic therapy alone, powder, bead, or pouch treatments displayed comparable mean *P. aeruginosa* levels, with the pouch treatment showing the lowest bioburden, which was statistically significantly lower than systemic therapy alone and bead treatments (*p* = 0.038 and *p* = 0.038; Table 2). Still, the absolute range of remaining *P. aeruginosa* bioburden differed by less than 1 log_10_ unit across all groups.

#### 3.4.3. Bioburden Ratios

**Figure 4:**
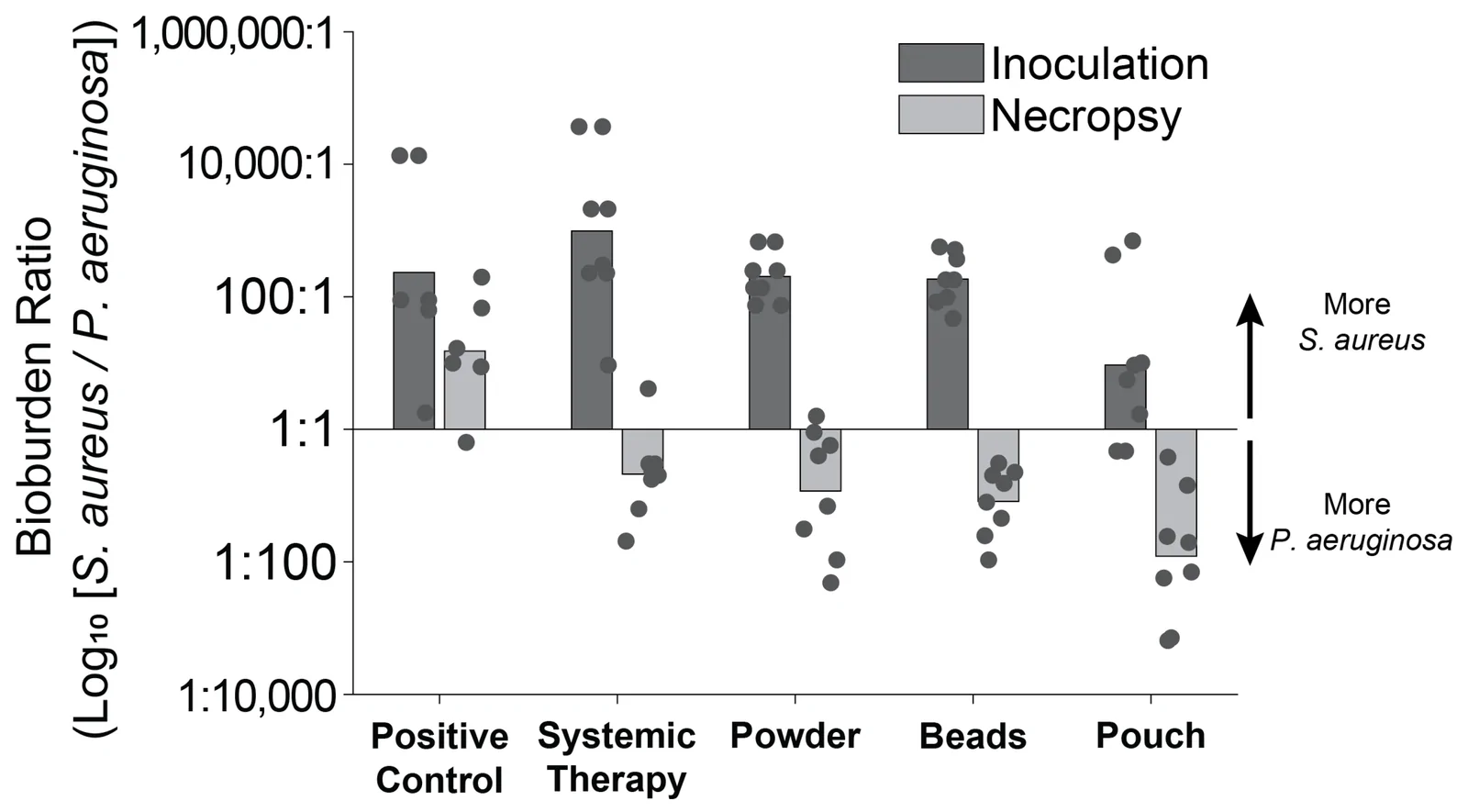
Ratio of *S. aureus* to *P. aeruginosa* bioburden across each treatment type. The figure shows the relative proportions of *S. aureus* and *P. aeruginosa* at the time of inoculation and in samples recovered at necropsy.

The ratio of *S. aureus* to *P. aeruginosa* shows a clear trend toward increased *P. aeruginosa* dominance following implantation, a shift that was further accentuated by antibiotic treatment (Figure 4). At inoculation, animals received a biofilm inoculum with an approximate average *S. aureus*:*P. aeruginosa* ratio of 200:1. Variability in reactor growth resulted in a higher ratio in the systemic therapy group (986:1) and a lower ratio in the pouch group (9:1). In samples collected at necropsy, the positive control group showed a modest shift toward *P. aeruginosa* while maintaining a greater relative abundance of *S. aureus* (15:1). In contrast, the systemic therapy (1:5), powder (1:9), bead (1:12), and pouch (1:82) groups exhibited pronounced decreases in the *S. aureus*:*P. aeruginosa* ratio, reflecting a substantial post-treatment shift in the microbiological balance favoring *P. aeruginosa*.

### 3.5. Histological Outcomes

**Figure 5:**
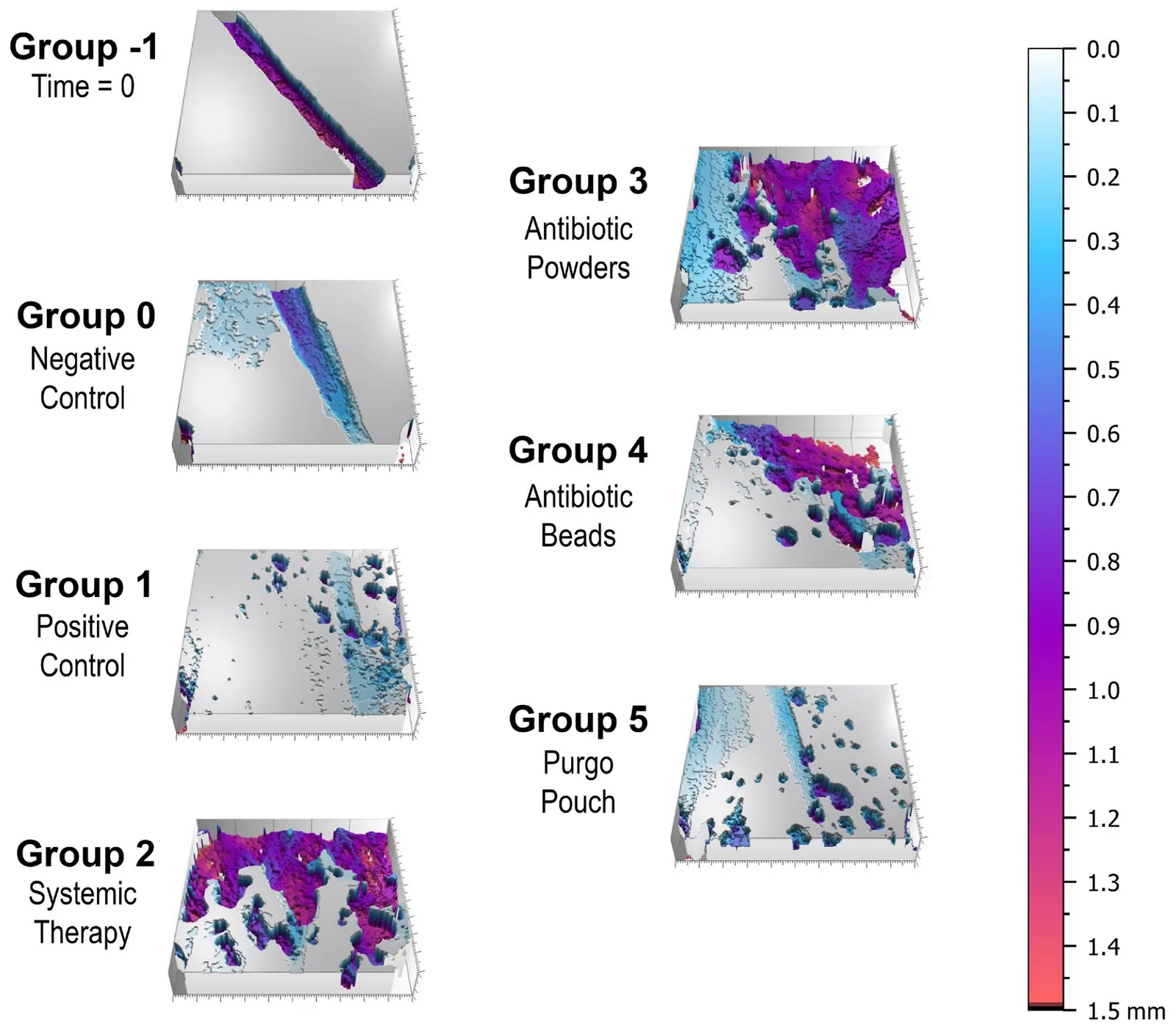
Representative 10 × 10 mm µ-CT 3D reconstructions of the osteotomized ROI below each plate. Resorption ranged up to a depth of 1.5 mm across samples (Z-axis). The Pouch (Group 5) showed a higher surface bone retention than other treatments. Major X-Y tick marks are 1 mm.

**Figure 6:**
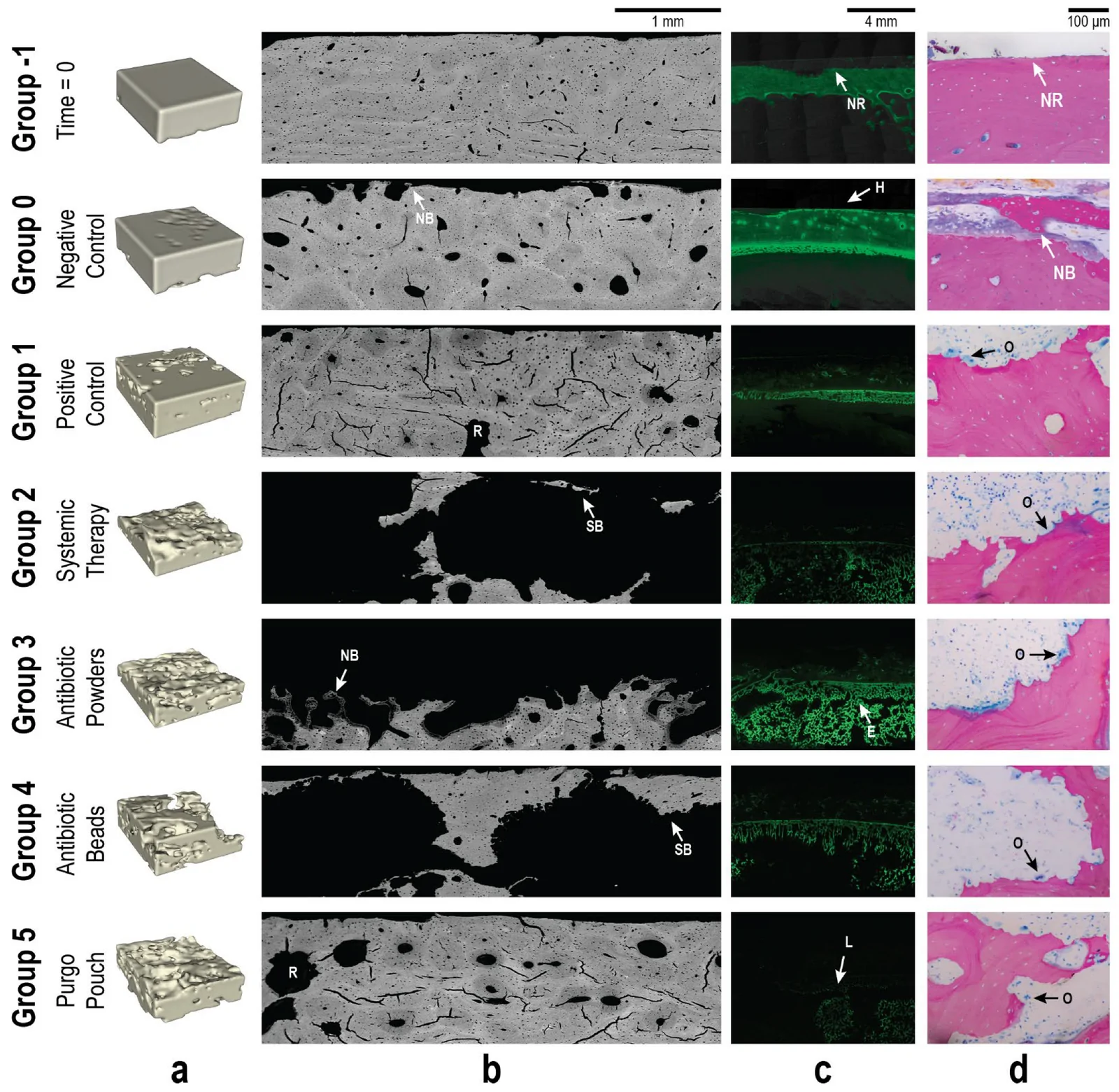
Representative qualitative histological outcomes. (**a**) µ-CT images of the extracted 4 × 4 mm ROI beneath the plate showed that the Pouch group (Group 5) retained more bone than systemic therapy alone (Group 2). (**b**) Backscattered SEM showed different bone responses. Higher levels of sequestrum bone (SB) were present in the systemic therapy (Group 2) and beads (Group 4) groups, whereas the Pouch (Group 5) showed only small pockets of resorption (R). Note the new bone (NB) formation under the plate in the antibiotic powder group (Group 3). (**c**) Fluorescent microscopy demonstrated uniform healthy remodeling (H) under the plate in the negative control specimens (Group 0). In contrast, a high amount of reactive bone in the endosteal region (E) was observed in the antibiotic powder group (Group 3). Limited remodeling (L) with no significant bone response (NR) was observed in the Time = 0 and Pouch groups (Groups -1 and 5, respectively). (**d**) Light microscopy showed NB with minimal osteoclast activity in the negative control group (Group 0). All other groups showed varying extents of osteoclasts (O) along resorption seams. SB = sequestrum bone, NB = new bone, H = healthy remodeling, NR = no response, E = endosteal reaction, L = limited remodeling, O = osteoclast.

Two previously published treatment groups were included for histological comparison in the datasets below [21]. Group -1 animals had histological analyses performed on naïve limb to establish baseline bone characteristics (time = 0), and Group 0 animals were a negative control group (no bacteria and 21-day endpoint) [21]. Both groups received the same surgical procedure as the animals described in this study. Additional statistical analyses are presented in Supplementary Materials. Representative histological images are shown (Figure 5 and Figure 6).

#### 3.5.1. Micro-Computed Tomography (µ-CT)

**Figure 7:**
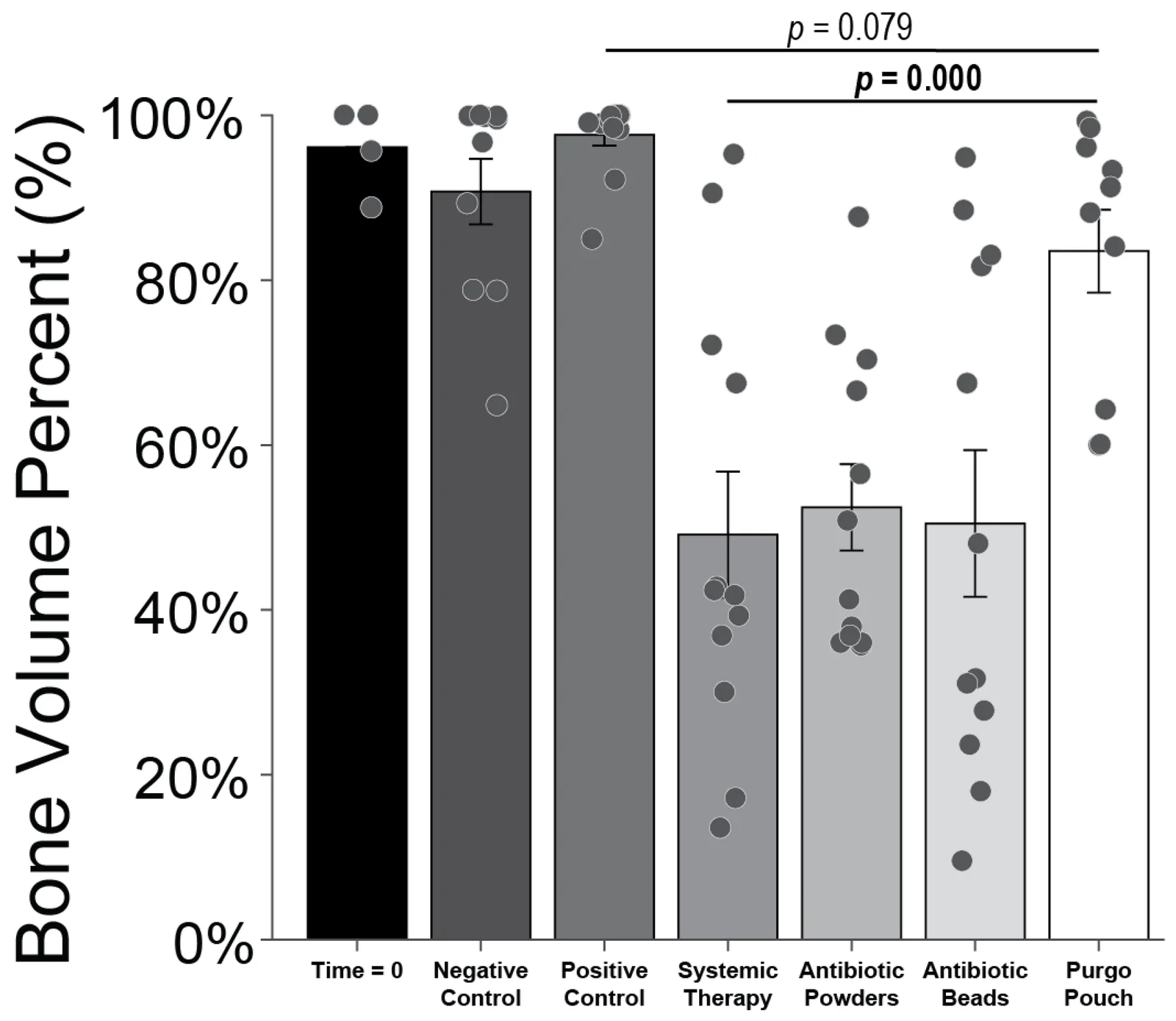
Bone volume percentage. The bone volume under the proximal fixation plate was determined using µ-CT. Group 5 (Purgo Pouch) had the highest bone volume among the antibiotic-treated groups. Statistical comparisons are reported in Tables S7 and S8. Samples are n = 4 at ‘time = 0’, n = 10 for the negative control, n = 12 for the positive control, systemic therapy alone, powders, antibiotic-loaded beads, and n = 10 for the pouch group, with two samples per animal analyzed. Outcomes were analyzed using a mixed-effects linear regression model, with *p*-values adjusted for multiple comparisons using a false discovery rate procedure (Benjamini–Krieger–Yekutieli). Error bars represent ± standard error.

μ-CT was used to evaluate the percentage of residual bone volume beneath the proximal fracture fixation plate (Figure 7). At baseline, animals in previously published findings exhibited a high baseline bone volume (96.1% ± 2.6%; Group -1), which was largely maintained in the negative control group after 21 days (90.7% ± 4.0%; Group 0) [21]. The slightly reduced bone volume in the negative controls compared to baseline was attributed to periosteum removal during surgery and the natural porosity of bone.

Infection generally led to a reduction in bone volume. Interestingly, the positive control animals (Group 1), which received no antibiotic treatment, exhibited bone volumes (97.6% ± 1.3%) comparable to baseline levels. In contrast, animals treated with systemic antibiotics alone, antibiotic powders, or antibiotic beads showed significantly lower bone volumes than the positive control group. Among the antibiotic-treated groups, the Purgo Pouch (Group 5) demonstrated the highest retained volume (83.5% ± 5.0%), showing a reduction in bone volume that was not statistically significant compared to the positive controls (*p* = 0.079; Table S7). Furthermore, Group 5 animals retained significantly more bone volume than those treated with systemic therapy alone (Group 2) (*p* < 0.001; Table S7).

#### 3.5.2. Scanning Electron Microscopy (SEM)

**Figure 8:**
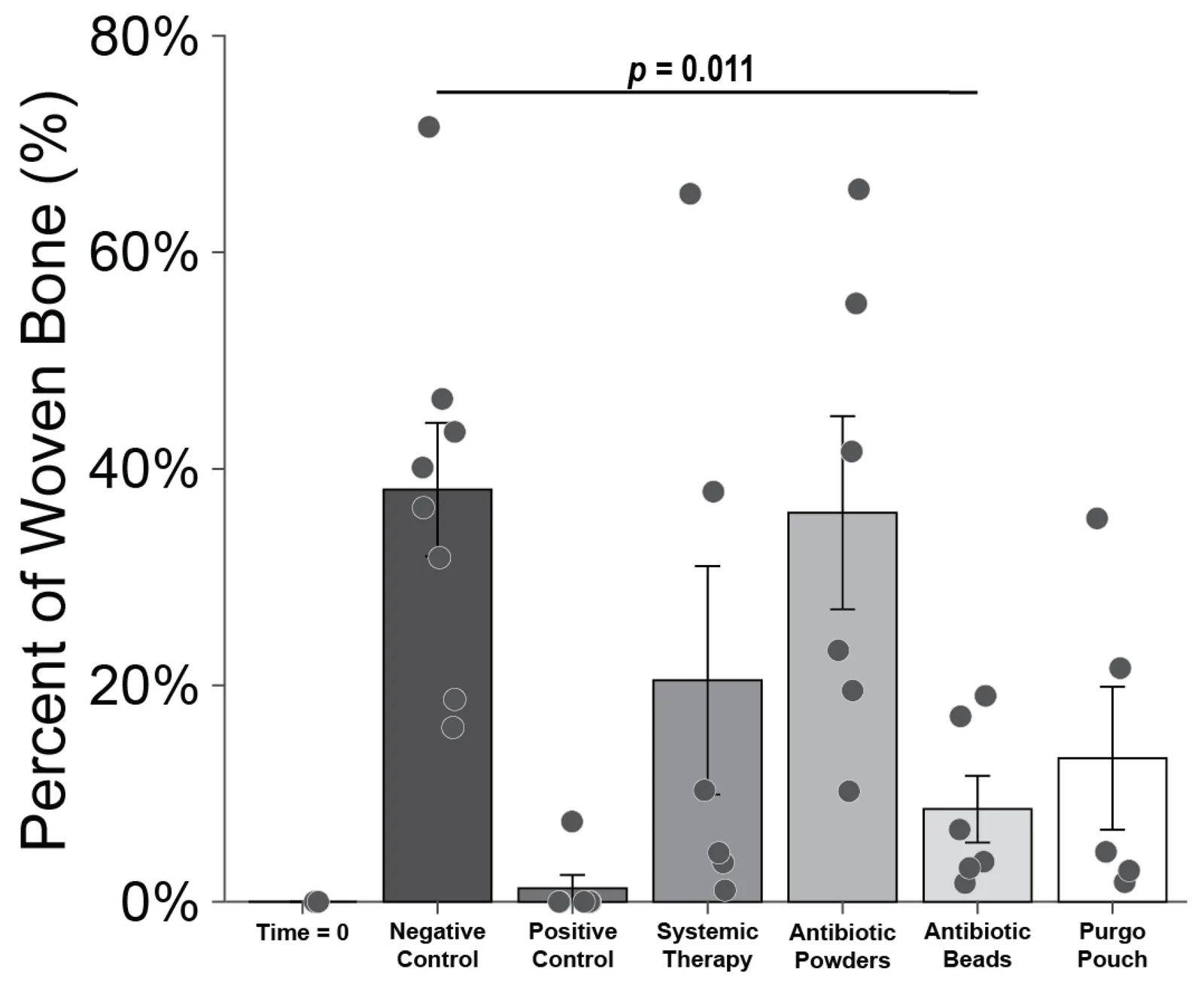
Woven bone percentage. The percentage of woven bone was evaluated using SEM. No comparisons between animals treated with antibiotics (Groups 2–5) were statistically significant. Statistical comparisons are reported in Tables S7 and S8. Samples are n = 2 at ‘time = 0’, n = 8 for the negative control, n = 6 for the positive control, systemic therapy alone, powders, antibiotic-loaded beads, and n = 5 for the pouch group. Group means were compared using two-sample *t*-tests with unequal variances (Welch *t*-tests), with *p*-values adjusted for multiple comparisons using a false discovery rate procedure (Benjamini–Krieger–Yekutieli). Error bars represent ± standard error.

SEM was used to quantify woven bone as a marker of new bone remodeling (Figure 8). As previously reported, no woven bone was present at baseline, as insufficient time had elapsed for healing [21]. In negative controls, substantial new bone formation (38.1% ± 6.2%) occurred in surgically affected regions, reflecting a natural response to periosteal removal, simulated fracture, and fixation plate placement. This confirmed that the surgical model did not impair normal bone healing.

Positive control animals (Group 1) showed a significantly reduced healing response compared to negative controls (Group 0) (*p* = 0.008; Table S7). The effects of antibiotic treatment varied by delivery method. Animals receiving antibiotic powders (Group 3) exhibited the greatest increase in woven bone toward baseline levels (35.9% ± 8.9%), which was statistically significant relative to positive controls (*p* = 0.037; Table S7). In contrast, antibiotic-loaded beads (Group 4) showed the poorest outcome (8.6% ± 3.1%), with significantly less woven bone than negative controls (*p* = 0.011; Table S7). No other differences between antibiotic-treated groups and controls were statistically significant.

#### 3.5.3. Light Microscopy

**Figure 9:**
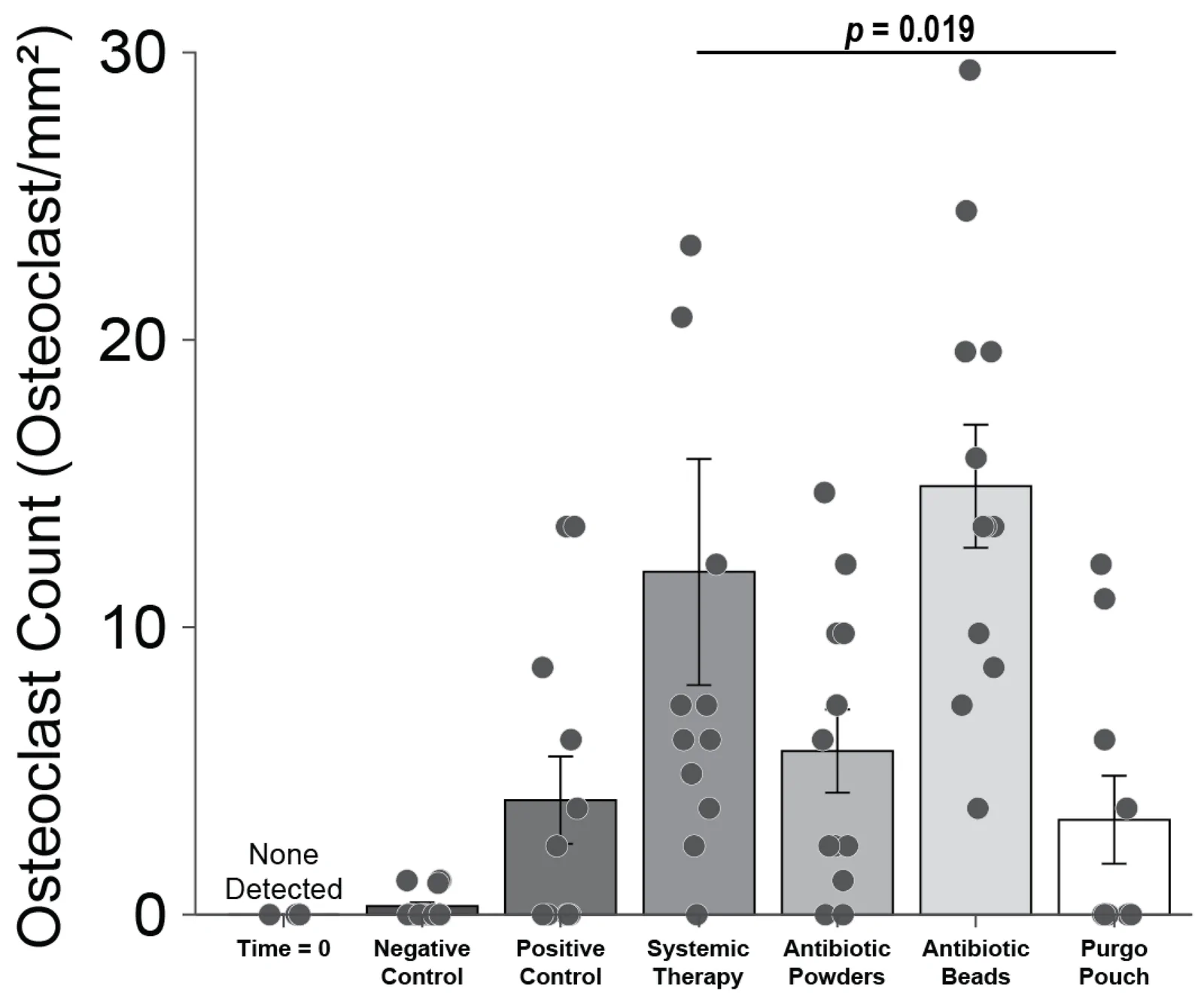
Osteoclast count. Light microscopy was used to count osteoclasts per mm^2^. Only Group 5 (Purgo Pouch) statistically reduced osteoclast presence compared to systemic therapy only (Group 2). Minimal differences were observed when antibiotic-loaded beads (Group 4) were added to systemic therapy alone (Group 2). One observation for the systemic therapy group had a value of 49.0, which is not on the plot but was used in all analyses. Statistical comparisons are reported in Tables S7 and S8. Samples are n = 4 at ‘time = 0’, n = 16 for the negative control, n = 12 for the positive control, systemic therapy alone, powders, antibiotic-loaded beads, and n = 10 for the pouch group, with two samples per animal analyzed. Outcomes were analyzed using a mixed-effects linear regression model, with *p*-values adjusted for multiple comparisons using a false discovery rate procedure (Benjamini–Krieger–Yekutieli). Error bars represent ± standard error.

Light microscopy was used to quantify osteoclast density (osteoclasts per mm^2^) (Figure 9). Previous data showed that at baseline, osteoclast activity was minimal (0.1 per mm^2^) and remained low in the negative control group (0.3 ± 0.1 per mm^2^) [21].

Infection was associated with elevated osteoclast activity. Positive control animals (Group 1) exhibited a modest increase in osteoclast density (4.0 ± 1.5 per mm^2^) compared to negative controls. Overall, the addition of antibiotic treatments appeared to amplify this response. Animals receiving systemic therapy alone (Group 2) showed a significant rise in osteoclast count (11.9 ± 3.9 per mm^2^) relative to positive controls (*p* = 0.020; Table S7), which further increased with the use of antibiotic beads (14.9 ± 2.1 per mm^2^). In contrast, osteoclast activity was markedly reduced in groups treated with antibiotic powders (Group 3) or the Purgo Pouch (Group 5), though only the Purgo Pouch group showed a statistically significant decrease compared to systemic therapy (*p* = 0.019; Table S7).

## 4. Discussion

Polymicrobial FRIs can pose treatment challenges that are fundamentally different from those of monomicrobial infections. Clinical studies consistently report that polymicrobial infections are associated with greater treatment recalcitrance, prolonged healing times, and higher recurrence rates [24]. Aspects of these challenges were observed in this study, with key takeaways being that no matter how antibiotics are delivered, *P. aeruginosa* can be particularly difficult to manage in this type of musculoskeletal infection, and if *S. aureus* within a polymicrobial system is killed to a greater degree than *P. aeruginosa*, *P. aeruginosa* can run away with infection if the antibiotic therapy is insufficient. But the observations were not solely related to antibiotic therapies. Interestingly, positive control animals (Group 1) exhibited a paradoxical outcome of host bone architecture remaining largely healthy despite the pathogenic inocula that contradicted our previous findings in monomicrobial studies, wherein bone health was severely compromised with high bacterial load [21]. The paradox was so unexpected that we performed work on n = 2 more positive control animals than we originally planned to confirm the outcome, and the result repeated. The results underscore the complexity of interpreting treatment success in the presence of polymicrobial, biofilm-driven infections.

Pathogen ratio data (Figure 4) demonstrated a clear trend towards *P. aeruginosa* in this polymicrobial model over a 21-day period, with positive-control animals shifting from a median ~200:1 ratio to 15:1 (*S. aureus*:*P. aeruginosa*) without antibiotic therapy. This shift in the absence of therapy is supported by several competitive mechanisms of increased virulence that favor *P. aeruginosa* in polymicrobial co-infection [27,35,36]. These mechanisms confer a progressive competitive advantage to *P. aeruginosa* over time and have been shown to inhibit *S. aureus* growth in both in vitro and in vivo systems, consistent with the ecological displacement observed in positive-control animals over the 21-day study period [37,38,39]. Against this backdrop of natural competitive dynamics, antibiotic exposure mixed with an 11-day gap between therapy and euthanasia appeared to further reshape the pathogen balance. The 10-day, broad-spectrum, biofilm-active systemic regimen (levofloxacin + rifampin; Group 2) failed to adequately control either species at the tissue, bone, or hardware level. Rifampin, particularly when combined with levofloxacin, is a potent agent for *S. aureus* biofilm-associated infections but has unreliable, regimen-dependent activity against *P. aeruginosa* at clinically relevant doses [40]. Bacterial imbalance was most dramatic in this group, in which *P. aeruginosa* hardware bioburden was reduced significantly less than in untreated animals, suggesting that systemic therapy disproportionately suppressed *S. aureus* while leaving *P. aeruginosa* relatively unchecked. In the absence of a locally applied antibacterial agent targeting both species, partial eradication of *S. aureus* driven by rifampin-containing systemic therapy may have further shifted the competitive balance towards *P. aeruginosa*. This concern is particularly relevant given the widespread use of rifampin as a biofilm-active Gram-positive agent, which may inadvertently skew the ecological niche toward the persistence or outgrowth of Gram-negative species, such as *P. aeruginosa*, when Gram-negative coverage is comparatively weaker.

The addition of adjuvant single-dose local delivery strategies (antibiotic powder (Group 3) or CaSO_4_ beads (Group 4)) provided inconsistent benefits and was associated with notable adverse effects on bone integrity. In contrast, reloading local antibiotic release via the Purgo Pouch (Group 5) preserved the highest bone volume of antibiotic-treated groups and was the only intervention to significantly reduce *S. aureus* tissue bioburden compared with the other treatments. But in all cases, the initial average inoculum ratio of approximately 200:1 (*S. aureus*:*P. aeruginosa*) exhibited an ecological shift toward *P. aeruginosa* dominance. Persistent *P. aeruginosa* colonization across all groups indicated that antibiotic selection pressures and time may influence interspecies interactions, i.e., if two antibiotics are both active against one of the polymicrobial isolates (vancomycin and tobramycin kill *S. aureus*) but only one is active against the other (only tobramycin is active against *P. aeruginosa*), and if local antibiotic therapy is discontinued too early (there was an 11-day gap between pouch removal and the endpoint), an imbalance may occur and the predominant organism may persist. This suggests that clinicians may need to consider regimen-driven shifts in microbial dominance when treating polymicrobial FRIs.

Notably, the shift towards *P. aeruginosa* predominance occurred across all antibiotic-treated groups. This finding is plausibly explained by the differential activity of the antibiotic regimen against the two species. Tobramycin and vancomycin, selected for their commonplace use in local orthopedic treatment (as powders or within carrier materials), showed a similar pattern of imbalance: tobramycin provides activity against both Gram-positive and Gram-negative organisms, including *P. aeruginosa* ATCC 27853 (MIC 0.125 µg/mL; laboratory testing), whereas vancomycin is largely ineffective against *P. aeruginosa* (MIC > 64 µg/mL; laboratory testing). Pouch-treated animals had the most pronounced shift towards *P. aeruginosa*, suggesting that the vancomycin/tobramycin combo readily killed *S. aureus*, but the tobramycin dose may have been too low to manage *P. aeruginosa*. However, the lower inoculation ratio makes direct comparisons difficult. While the pouch animals recovered the lowest absolute *P. aeruginosa* bioburden, the absolute difference in less than 0.5 log_10_ units is likely not clinically meaningful. These observations may have important clinical implications: broad-spectrum or combination antibiotic coverage that nominally “covers all organisms” does not guarantee balanced suppression of all pathogens within a polymicrobial biofilm, and the duration of sustained local therapy likely matters. Dosing and duration optimization are warranted and may more effectively address the previously described synergy of polymicrobial biofilms [39,41] and competitive interactions that can increase antimicrobial tolerance [42].

Importantly, the histological outcomes of this study indicated that polymicrobial infections cannot be thought of solely through a microbiological lens. Positive control animals (Group 1), which harbored the greatest collective bioburden, paradoxically retained bone volumes comparable to baseline and exhibited lower osteoclast activity than any antibiotic-treated group. Previous investigations using this ovine model with monomicrobial *S. aureus*, MRSA, and *P. aeruginosa* biofilms consistently demonstrated significant bone loss, minimal woven bone formation, and marked osteoclast presence in positive controls; features characteristic of a robust osteolytic infection response [5,21]. Yet the distinct pattern observed here was likely attributable to the pathogen ratio, as the positive control was the only group to maintain *S. aureus* dominance over *P. aeruginosa* at necropsy (15:1). A competitive equilibrium between the two species may have attenuated the virulence of each, thereby diminishing the net osteolytic response. By contrast, as antibiotic therapies cleared *S. aureus* to a greater degree, osteoclastogenesis may have been stimulated via inflammatory debris and permitted *P. aeruginosa* overgrowth, together amplifying bone resorption [43]. Systemic therapy alone appeared to exacerbate negative bone outcomes, dramatically reducing bone volume while increasing osteoclast proliferation. The addition of adjuvant local therapy had mixed efficacy in rescuing bone integrity, with outcomes varying markedly by carrier.

Antibiotic-loaded CaSO_4_ beads (Group 4) resulted in poor histological findings, including low bone volume, minimal woven bone, and a high osteoclast count, suggesting limited resolution of the ongoing infection. This mirrors previous work in this ovine model, where bead use was associated with adverse histological findings, and aligns with clinical evidence that bead byproducts can create an acidic microenvironment with osteolytic effects [21,44]. Animals treated with antibiotic powders (Group 3) exhibited high woven bone, a robust endosteal reaction, and significant bone volume loss, a pattern consistent with active but dysregulated repair in the setting of residual infection. Clinically, a high degree of bone loss and reactive new bone formation, alongside unresolved infection, may indicate a surgical site at risk for non-union or requiring revision [45]. By contrast, the Purgo Pouch (Group 5) group demonstrated low woven bone, low osteoclast activity, and the best-preserved bone volume among antibiotic-treated groups, suggesting a more quiescent bone environment potentially driven by improved *S. aureus* suppression or the sustained presence of local antibiotics. Notably, this bone preservation was achieved without the reactive new bone formation observed in the powder group, which may suggest more sustained infection control. These data suggest that reloading local antibiotic therapy may be an important determinator of bone response, even under similar systemic regimens. Importantly, serum antibiotic concentrations for all local delivery groups remained at or below the limit of detection, suggesting that the histological differences observed between local strategies reflect local effects rather than differences in systemic exposure.

While polymicrobial biofilm infections are common in musculoskeletal infections clinically, in vivo animal models that combine polymicrobial biofilm inoculation of bone or orthopedic implants with systemic or local antibiotic treatment are scarce and largely restricted to soft tissue or wound models [46,47,48]. The data here demonstrated that the interspecies ecology of *S. aureus* and *P. aeruginosa* co-infection introduces treatment dynamics that are not captured in single-organism models and may not be adequately addressed by existing strategies. The positive control data suggested that an undisturbed polymicrobial biofilm in competitive equilibrium may be less initially osteolytic than a partially treated one, a pattern not observed in prior monomicrobial work with this model [5,21], and, to our knowledge, is not commonly reported. If confirmed in future polymicrobial studies, and without attempting to overstate considerations based on a single study, the data suggested that clinically, it may be important for the threshold of antibiotic treatment in polymicrobial FRI to match the capacity to sustain and complete it; a treatment course that partially suppresses *S. aureus* without adequately controlling *P. aeruginosa* may shift the infection toward a more bone-destructive Gram-negative-dominant state that is worse than the pre-treatment equilibrium. Optimized local delivery systems for polymicrobial infections represent one potential treatment modality. These data also indicated that bioburden reduction and bone preservation are not interchangeable outcomes in polymicrobial FRI; treatments achieving equivalent or even superior microbiological results can yield inferior bone outcomes, which may depend on local antibiotics and organism dominance. While potentially impactful, these observations must be interpreted in the context of several important study limitations.

The translatability of this study is limited by considerations that motivate future research. First, variability in the inoculation ratio across treatment groups complicates direct between-group comparisons of pathogen ratios. The oral systemic regimen of levofloxacin and rifampin aligns with prior work and a plausible clinical dosing strategy but may have limited bioavailability in the ovine model due to the ruminant digestive tract. Further polymicrobial studies using alternative dosing regimens, including intravenous delivery and agents such as ciprofloxacin, which has excellent activity against *P. aeruginosa*, are necessary to clarify the biofilm response to systemic treatment. Additional work is also needed to characterize microbiological and histological patterns beyond the single temporal snapshot of 10-day treatment with a 21-day endpoint. A multi-timepoint euthanasia model would help determine whether microbiological trends and histological patterns (quiescence, reactive formation, active resorption) represent different stages of a shared temporal trajectory of infection resolution, or fundamentally different healing outcomes. A longer follow-up would also help elucidate whether the shift toward *P. aeruginosa* in untreated controls ultimately reaches equilibrium or progresses to complete dominance, and whether this outcome eventually results in histological dysregulation once co-competitive forces between the species are resolved.

Further tobramycin dose-escalation studies using local delivery to target *P. aeruginosa* bioburden may help define the histological consequences of maintaining the species ratio throughout treatment. While reloadability is a key feature of the Pouch device, repeated dosing resulted in greater cumulative antibiotic exposure and a longer effective treatment duration than the single-dose powder and CaSO_4_ bead strategies. Consequently, the present study cannot isolate the independent contributions of cumulative antibiotic dose, dosing duration, reloading frequency, local release kinetics, and carrier biology to the observed microbiological and bone outcomes. Future dose-matched comparative studies are needed to isolate the effects of reloadable sustained local delivery from those of total antibiotic exposure. This investigation raises important questions for future work about how to optimize antibiotic selection, dosing route, and delivery kinetics to balance microbiological control with preservation of bone integrity in polymicrobial FRI. Future work could also address whether two or more antibiotics loaded into the pouch, all active against *S. aureus* and *P. aeruginosa*, would improve outcomes.

## 5. Conclusions

In broad terms, these data demonstrated that polymicrobial *S. aureus* and *P. aeruginosa* FRIs may pose a treatment challenge distinct from monomicrobial infection, defined not only by the difficulty of eradicating two organisms simultaneously but also by the potential ecological dynamics between them. In this investigation, a broad-spectrum, biofilm-active systemic regimen appeared to amplify a natural shift toward *P. aeruginosa* dominance rather than suppressing it, and single-bolus local strategies provided inconsistent microbiological benefit with notable adverse bone consequences. Reloading local antibiotic delivery via the pouch best preserved skeletal integrity and achieved the greatest *S. aureus* tissue reduction, but universal *P. aeruginosa* tissue persistence across all groups identified Gram-negative coverage as a significant consideration.

Taken together, these findings support a paradigm in which successful management of polymicrobial FRIs will require not only potent biofilm-active agents, but also ecologically informed combination strategies that prevent pathogen replacement and ensure simultaneous, durable suppression of organisms. The outcomes highlight the clinical implications and the potential limitations of current therapeutic approaches for polymicrobial FRIs, while demonstrating that reloadable, local therapy shows promise.

## Figures and Tables

**Table 1:** Animal Treatment Groups. A description of the animal groups and their antimicrobial treatment in the right hind limb adjacent to the biofilm-ridden simulated fracture fixation plates. PO = by mouth; QD = once daily. The Purgo Pouch is refillable, enabling greater total local antibiotic exposure over the treatment period than non-reloadable strategies.

Animal Group	Group Shorthand	Bacteria	Treatment	Total Local Antibiotic Exposure	Endpoint
Group 1 (n = 6)	Positive Control	*S. aureus* and *P. aeruginosa*	None	None	21 Days
Group 2 (n = 8)	Systemic Therapy	*S. aureus* and *P. aeruginosa*	750 mg Levofloxacin and 600 mg Rifampin PO QD for 10 days	None	21 Days
Group 3 (n = 8)	Powder	*S. aureus* and *P. aeruginosa*	750 mg Levofloxacin and 600 mg Rifampin PO QD for 10 days + 1 g vancomycin and 1.2 g tobramycin poured into the surgical site before closure	1 g Vancomycin + 1.2 g Tobramycin	21 Days
Group 4 (n = 8)	Beads	*S. aureus* and *P. aeruginosa*	750 mg Levofloxacin and 600 mg Rifampin PO QD for 10 days + calcium sulfate beads loaded with 1 g vancomycin and 240 mg tobramycin added to the surgical site before closure	1 g Vancomycin + 240 mg Tobramycin	21 Days
Group 5 (n = 8)	Pouch	*S. aureus* and *P. aeruginosa*	750 mg Levofloxacin and 600 mg Rifampin PO QD for 10 days + the Purgo Pouch emptied and reloaded QD with a solution containing 500 mg vancomycin and 240 mg of tobramycin for 10 days	5 g Vancomycin + 2.4 g Tobramycin	21 Days

**Table 2:** Microbiology Statistical Comparisons. Comparisons between individual samples were performed with a Welch’s *t*-test (unequal variance). Group 1 = Positive Control; Group 2 = Systemic Therapy; Group 3 = Powder; Group 4 = Beads; Group 5 = Pouch. *p*-values were adjusted using a Benjamini–Krieger–Yekutieli two-stage step-up false discovery rate (FDR) procedure to limit the false-positive findings (Type I errors). Statistically significant comparisons (*p* < 0.05) are bold.

		Group Comparison									
Analysis		1 vs. 5	1 vs. 4	1 vs. 3	1 vs. 2	2 vs. 5	2 vs. 4	2 vs. 3	3 vs. 5	3 vs. 4	4 vs. 5
3.4.1.A: *S. aureus* Hardware Reduction Scores	*p*-value	**0.005**	**<0.001**	**0.003**	**0.008**	0.394	0.253	0.341	0.959	0.961	0.988
	Adjusted *p*-value	**0.011**	**<0.001**	**0.010**	**0.013**	0.355	0.319	0.355	0.622	0.622	0.622
3.4.1.B: *P. aeruginosa* Hardware Reduction Scores	*p*-value	0.292	0.665	0.571	**0.017**	**<0.001**	**0.009**	**0.006**	**0.006**	0.892	**0.042**
	Adjusted *p*-value	0.219	0.388	0.375	**0.018**	**<0.001**	**0.012**	**0.011**	**0.011**	0.468	**0.037**
3.4.2.A: *S. aureus* Tissue Bioburden	*p*-value	**<0.001**	**<0.001**	**0.001**	**<0.001**	**0.001**	0.095	0.249	**0.043**	0.842	**0.026**
	Adjusted *p*-value	**<0.001**	**<0.001**	**0.001**	**<0.001**	**0.001**	0.050	0.116	**0.026**	0.354	**0.018**
3.4.2.B: *P. aeruginosa* Tissue Bioburden	*p*-value	0.116	0.892	0.312	0.727	**0.006**	0.461	0.147	0.797	0.271	**0.009**
	Adjusted *p*-value	0.309	0.749	0.437	0.744	**0.038**	0.553	0.309	0.744	0.437	**0.038**

## Data Availability

The datasets generated and/or analyzed during the current study are not publicly available due to an active FDA submission, but may be available from the corresponding authors on reasonable request.

## Supplementary Materials

The following supporting information can be downloaded at https://www.mdpi.com/article/10.3390/microorganisms14092062/s1, Figure S1: *Staphylococcus aureus* individual sample bioburden. The average *S. aureus* bioburden (log10 units) recovered, broken down by sample. Hardware samples are reported as the total bioburden collected per sample or set of samples (the four screw samples are aggregated), and tissue samples are normalized by weight. Statistical comparisons between the samples are reported in Table S1. Error bars represent ± standard error.; Figure S2: *Pseudomonas aeruginosa* individual sample bioburden. The average *P. aeruginosa* bioburden (log_10_ units) recovered, broken down by sample. Hardware samples are reported as the total bioburden collected per sample or set of samples (the four screw samples are aggregated), and tissue samples are normalized by weight. Statistical comparisons between the samples are reported in Table S3. Error bars represent ± standard error; Figure S3: Combined *Staphylococcus aureus* and *Pseudomonas aeruginosa* sample bioburden. For each sample, the reported value is the average bioburden (log_10_ units) calculated from the combined recovery of *S. aureus* and *P. aeruginosa*. Hardware samples are reported as the total bioburden collected per sample or set of samples (the four screw samples are aggregated), and tissue samples are normalized by weight. Statistical comparisons between the samples are reported in Table S5 below. Error bars represent ± standard error. Table S1: *Staphylococcus aureus* microbiology statistical comparisons adjusted with an FDR procedure. Group 1 = Pos. Ctrl.; Group 2 = Systemic Therapy; Group 3 = Antibiotic Powders; Group 4 = Antibiotic Beads; Group 5 = Pouch. Group means were compared using two-sample *t*-tests with unequal variances (Welch *t*-tests. *p*-values were adjusted using a Benjamini-Krieger-Yekutieli two-stage step-up false discovery rate (FDR) procedure to limit the false-positive findings (Type I errors). Statistically significant comparisons (*p* < 0.05) are bold; Table S2: *Staphylococcus aureus* unadjusted microbiology statistical comparisons. Group 1 = Pos. Ctrl.; Group 2 = Systemic Therapy; Group 3 = Antibiotic Powders; Group 4 = Antibiotic Beads; Group 5 = Pouch. Group means were compared using two-sample *t*-tests with unequal variances (Welch *t*-tests). Statistically significant comparisons (*p* < 0.05) are bold; Table S3: *Pseudomonas aeruginosa* microbiology statistical comparisons adjusted with an FDR procedure. Group 1 = Pos. Ctrl.; Group 2 = Systemic Therapy; Group 3 = Antibiotic Powders; Group 4 = Antibiotic Beads; Group 5 = Pouch. Group means were compared using two-sample *t*-tests with unequal variances (Welch *t*-tests). *p*-values were adjusted using a Benjamini-Krieger-Yekutieli two-stage step-up false discovery rate (FDR) procedure to limit the false-positive findings (Type I errors). Statistically significant comparisons (*p* < 0.05) are bold; Table S4: *Pseudomonas aeruginosa* unadjusted microbiology statistical comparisons. Group 1 = Pos. Ctrl.; Group 2 = Systemic Therapy; Group 3 = Antibiotic Powders; Group 4 = Antibiotic Beads; Group 5 = Pouch. Group means were compared using two-sample *t*-tests with unequal variances (Welch *t*-tests). Statistically significant comparisons (*p* < 0.05) are bold; Table S5: Combined *Staphylococcus aureus* and *Pseudomonas aeruginosa* microbiology statistical comparisons adjusted with an FDR procedure. Group 1 = Pos. Ctrl.; Group 2 = Systemic Therapy; Group 3 = Antibiotic Powders; Group 4 = Antibiotic Beads; Group 5 = Pouch. Group means were compared using two-sample *t*-tests with unequal variances (Welch *t*-tests). *p*-values were adjusted using a Benjamini-Krieger-Yekutieli two-stage step-up false discovery rate (FDR) procedure to limit the false-positive findings (Type I errors). Statistically significant comparisons (*p* < 0.05) are bold; Table S6: Combined *Staphylococcus aureus* and *Pseudomonas aeruginosa* unadjusted microbiology statistical comparisons. Group 1 = Pos. Ctrl.; Group 2 = Systemic Therapy; Group 3 = Antibiotic Powders; Group 4 = Antibiotic Beads; Group 5 = Pouch. Group means were compared using two-sample *t*-tests with unequal variances (Welch *t*-tests). Statistically significant comparisons (*p* < 0.05) are bold; Table S7: Histological statistical comparisons adjusted with an FDR procedure. The woven bone samples were compared with Welch’s *t*-tests (unequal variance), and the osteoclast and bone volume samples were compared using mixed-effect linear regression models. Group -1 = Time = 0; Group 0 = Negative Control; Group 1 = Positive Control; Group 2 = Systemic Therapy; Group 3 = Antibiotic Powders; Group 4 = Antibiotic Beads; Group 5 = Pouch. *p*-values were adjusted using a Benjamini-Krieger-Yekutieli two-stage step-up false discovery rate (FDR) procedure to limit the false-positive findings (Type I errors). Statistically significant comparisons (*p* < 0.05) are bold; Table S8: Histological unadjusted statistical comparisons. The woven bone samples were compared with Welch’s *t*-tests (unequal variance), and the osteoclast and bone volume samples were compared using mixed-effect linear regression models. Group -1 = Time = 0; Group 0 = Negative Control; Group 1 = Positive Control; Group 2 = Systemic Therapy; Group 3 = Antibiotic Powders; Group 4 = Antibiotic Beads; Group 5 = Pouch. Statistically significant comparisons (*p* < 0.05) are bold; Table S9: Effect size for S. aureus hardware reduction scores. Group 1 = Positive Control; Group 2 = Systemic Therapy; Group 3 = Antibiotic Powders; Group 4 = Antibiotic Beads; Group 5 = Pouch. comparisons performed using Welch’s two-sample *t*-test. Cohen’s d was calculated using the pooled standard deviations. Effect size benchmarks: negligible d < 0.2, small d = 0.2, medium d = 0.5, large d ≥ 0.8. Group A refers to the first group in the group comparison column; Group B refers to the second group in the group comparison column; Table S10: Effect size for *P. aeruginosa* hardware reduction scores. Group 1 = Positive Control; Group 2 = Systemic Therapy; Group 3 = Antibiotic Powders; Group 4 = Antibiotic Beads; Group 5 = Pouch. comparisons performed using Welch’s two-sample *t*-test. Cohen’s d was calculated using the pooled standard deviations. Effect size benchmarks: negligible d < 0.2, small d = 0.2, medium d = 0.5, large d ≥ 0.8. Group A refers to the first group in the group comparison column; Group B refers to the second group in the group comparison column; Table S11: Effect size for *S. aureus* tissue bioburden. Group 1 = Positive Control; Group 2 = Systemic Therapy; Group 3 = Antibiotic Powders; Group 4 = Antibiotic Beads; Group 5 = Pouch. comparisons performed using Welch’s two-sample *t*-test. Cohen’s d was calculated using the pooled standard deviations. Effect size benchmarks: negligible d < 0.2, small d = 0.2, medium d = 0.5, large d ≥ 0.8. Group A refers to the first group in the group comparison column; Group B refers to the second group in the group comparison column; Table S12: Effect size for *P. aeruginosa* tissue bioburden. Group 1 = Positive Control; Group 2 = Systemic Therapy; Group 3 = Antibiotic Powders; Group 4 = Antibiotic Beads; Group 5 = Pouch. comparisons performed using Welch’s two-sample *t*-test. Cohen’s d was calculated using the pooled standard deviations. Effect size benchmarks: negligible d < 0.2, small d = 0.2, medium d = 0.5, large d ≥ 0.8. Group A refers to the first group in the group comparison column; Group B refers to the second group in the group comparison column.
