# Supplementary material for: Antibiotic Selection for Sustained Local Therapy May Be Important to Reduce Pseudomonas aeruginosa Dominance Against Staphylococcus aureus and Bone Response Modulation in an Ovine Polymicrobial Fracture-Related Infection Model
Source: Microorganisms. 2026 Sep 16;14(9):2062. doi: 10.3390/microorganisms14092062 (PMC13609462; doi:10.3390/microorganisms14092062)
Supplement: Supplementary file 1 [file microorganisms-14-02062-s001.zip › microorganisms-4425562-supplementary.pdf]

microorganisms

MDPI

Supplementary Materials: Supplemental statistics and analyses

1 Supplemental microbiology

1.1 Staphylococcus aureus microbiology

Animals were inoculated with a consistent *S. aureus* bioburden, with no statistically significant differences between groups (Table S1). Overall, animals receiving adjuvant local therapy recovered less bioburden than those treated with systemic therapy alone (Fig. S-1), with pouch-treated animals (Group 5) showing the lowest residual bioburden across all sample types.

On hardware, all treated groups had significantly lower *S. aureus* recovery than the positive control for both screws and fixation plates (Table S1). Although pouch-treated animals recovered the lowest bioburden among treatment groups, differences between local therapies and systemic therapy were not statistically significant.

A similar pattern was observed in tissue samples. Local therapies outperformed systemic therapy overall, with pouch-treated animals again demonstrating the lowest bioburden. In soft tissue, Group 5 had significantly lower bioburden than the systemic therapy group (Group 2) (p=0.009; Table S1). In bone marrow, all antibiotic-treated groups (Groups 2–5) significantly outperformed the positive control, but no additional between-group differences reached significance. For screw track samples, the only significant difference was a reduction in bioburden in pouch-treated animals compared to the positive control (p=0.000; Table S1). No significant differences were observed between groups in bone samples (Table S1).

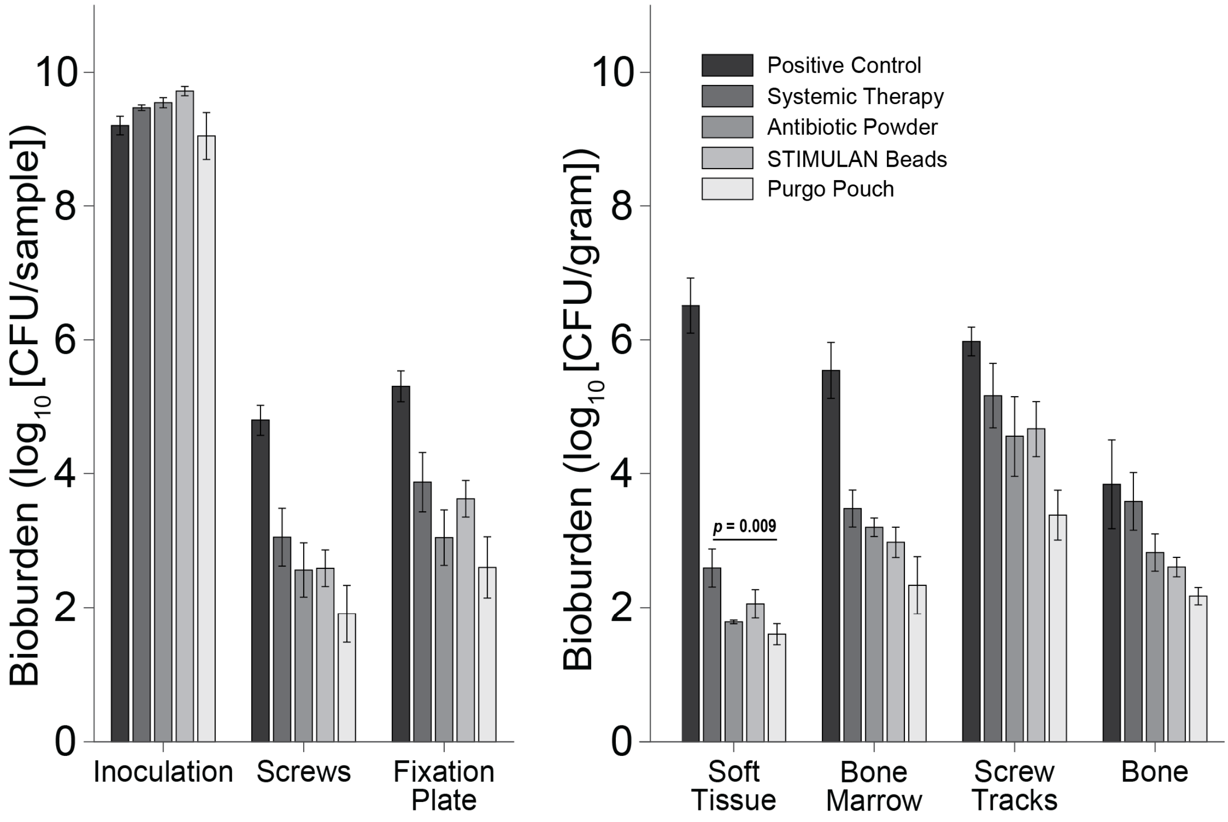

Figure S1. *Staphylococcus aureus* individual sample bioburden. The average *S. aureus* bioburden (log10 units) recovered, broken down by sample. Hardware samples are reported as the total bioburden collected per sample or set of samples (the four screw samples are aggregated), and tissue

samples are normalized by weight. Statistical comparisons between the samples are reported in Table S1. Error bars represent ± standard error.

Table S1: ***Staphylococcus aureus*** **microbiology statistical comparisons adjusted with an FDR procedure**. Group 1 = Pos. Ctrl.; Group 2 = Systemic Therapy; Group 3 = Antibiotic Powders; Group 4 = Antibiotic Beads; Group 5 = Pouch. Group means were compared using two-sample *t*-tests with unequal variances (Welch *t*-tests. *p*-values were adjusted using a Benjamini-Krieger-Yekutieli two-stage step-up false discovery rate (FDR) procedure to limit the false-positive findings (Type I errors). Statistically significant comparisons ($p < 0.05$) are bold.

**Inoculation**		**Screws**		**Fixation Plate**		**Soft Tissue**		**Bone Marrow**		**Screw Tracks**		**Bone**	
**Group Compar-ison**	**Adjusted *p*-value**	**Group Comparison**	**Adjusted *p*-value**	**Group Comparison**	**Adjusted *p*-value**	**Group Comparison**	**Adjusted *p*-value**	**Group Comparison**	**Adjusted *p*-value**	**Group Comparison**	**Adjusted *p*-value**	**Group Comparison**	**Ad-juste d *p*-value**
1 vs. 5	0.724	1 vs. 5	**0.000**	1 vs. 5	**0.000**	1 vs. 5	**0.000**	1 vs. 5	**0.000**	1 vs. 5	**0.000**	1 vs. 5	0.128
1 vs. 4	0.063	1 vs. 4	**0.000**	1 vs. 4	**0.002**	1 vs. 4	**0.000**	1 vs. 4	**0.003**	1 vs. 4	0.057	1 vs. 4	0.215
1 vs. 3	0.203	1 vs. 3	**0.002**	1 vs. 3	**0.002**	1 vs. 3	**0.000**	1 vs. 3	**0.004**	1 vs. 3	0.100	1 vs. 3	0.264
1 vs. 2	0.203	1 vs. 2	**0.008**	1 vs. 2	**0.025**	1 vs. 2	**0.000**	1 vs. 2	**0.005**	1 vs. 2	0.213	1 vs. 2	0.793
2 vs. 5	0.353	2 vs. 5	0.101	2 vs. 5	0.083	2 vs. 5	**0.009**	2 vs. 5	0.055	2 vs. 5	0.057	2 vs. 5	0.128
2 vs. 4	0.063	2 vs. 4	0.295	2 vs. 4	0.405	2 vs. 4	0.082	2 vs. 4	0.162	2 vs. 4	0.468	2 vs. 4	0.128
2 vs. 3	0.477	2 vs. 3	0.295	2 vs. 3	0.175	2 vs. 3	**0.018**	2 vs. 3	0.261	2 vs. 3	0.468	2 vs. 3	0.245
3 vs. 5	0.308	3 vs. 5	0.257	3 vs. 5	0.339	3 vs. 5	0.121	3 vs. 5	0.092	3 vs. 5	0.191	3 vs. 5	0.128
3 vs. 4	0.203	3 vs. 4	0.603	3 vs. 4	0.209	3 vs. 4	0.114	3 vs. 4	0.261	3 vs. 4	0.834	3 vs. 4	0.590
4 vs. 5	0.203	4 vs. 5	0.213	4 vs. 5	0.084	4 vs. 5	0.066	4 vs. 5	0.167	4 vs. 5	0.087	4 vs. 5	0.128

Table S2: *Staphylococcus aureus* unadjusted microbiology statistical comparisons. Group 1 = Pos. Ctrl.; Group 2 = Systemic Therapy; Group 3 = Antibiotic Powders; Group 4 = Antibiotic Beads; Group 5 = Pouch. Group means were compared using two-sample *t*-tests with unequal variances (Welch *t*-tests). Statistically significant comparisons ($p < 0.05$) are bold.

Inoculation		Screws		Fixation Plate		Soft Tissue		Bone Marrow		Screw Tracks		Bone	
Group Comparison	Adjusted *p*-value	Group Comparison	Adjusted *p*-value	Group Comparison	Adjusted *p*-value	Group Comparison	Adjusted *p*-value	Group Comparison	Adjusted *p*-value	Group Comparison	Adjusted *p*-value	Group Comparison	Adjusted *p*-value
1 vs. 5	0.689	1 vs. 5	**0.000**	1 vs. 5	**0.000**	1 vs. 5	**0.000**	1 vs. 5	**0.000**	1 vs. 5	**0.000**	1 vs. 5	0.053
1 vs. 4	**0.012**	1 vs. 4	**0.000**	1 vs. 4	**0.001**	1 vs. 4	**0.000**	1 vs. 4	**0.001**	1 vs. 4	**0.018**	1 vs. 4	0.123
1 vs. 3	0.063	1 vs. 3	**0.001**	1 vs. 3	**0.001**	1 vs. 3	**0.000**	1 vs. 3	**0.002**	1 vs. 3	0.053	1 vs. 3	0.201
1 vs. 2	0.116	1 vs. 2	**0.005**	1 vs. 2	**0.016**	1 vs. 2	**0.000**	1 vs. 2	**0.003**	1 vs. 2	0.158	1 vs. 2	0.755
2 vs. 5	0.269	2 vs. 5	0.080	2 vs. 5	0.066	2 vs. 5	**0.011**	2 vs. 5	**0.044**	2 vs. 5	**0.012**	2 vs. 5	**0.013**
2 vs. 4	**0.010**	2 vs. 4	0.382	2 vs. 4	0.642	2 vs. 4	0.156	2 vs. 4	0.180	2 vs. 4	0.446	2 vs. 4	0.061
2 vs. 3	0.409	2 vs. 3	0.422	2 vs. 3	0.194	2 vs. 3	**0.025**	2 vs. 3	0.385	2 vs. 3	0.442	2 vs. 3	0.163
3 vs. 5	0.205	3 vs. 5	0.285	3 vs. 5	0.484	3 vs. 5	0.288	3 vs. 5	0.088	3 vs. 5	0.121	3 vs. 5	0.061
3 vs. 4	0.111	3 vs. 4	0.957	3 vs. 4	0.265	3 vs. 4	0.245	3 vs. 4	0.414	3 vs. 4	0.883	3 vs. 4	0.506
4 vs. 5	0.100	4 vs. 5	0.203	4 vs. 5	0.080	4 vs. 5	0.110	4 vs. 5	0.212	4 vs. 5	**0.037**	4 vs. 5	**0.043**

1.2 Pseudomonas aeruginosa microbiology

Inoculation with *P. aeruginosa* demonstrated greater variability across groups. Notably, Group 5 (pouch) animals received significantly higher initial inocula than all groups except the positive control. Animals treated with local therapy generally exhibited lower recovered bioburden than those receiving systemic therapy alone. The positive control group showed a divergent pattern, with the lowest bioburden on hardware and screw track samples, but the highest in soft tissue and bone marrow.

On hardware, although the positive control group had the lowest recovered bioburden, no between-group comparisons reached statistical significance (Table S3). In soft tissue, all local therapy groups had significantly lower bioburden than the systemic therapy group (Group 2), with no significant differences among local treatments.

In bone marrow, all antibiotic-treated groups (Groups 2–5) demonstrated significantly lower bioburden than the positive control. However, pouch-treated animals (Group 5) showed a nonsignificant greater amount of recovered bioburden compared with systemic therapy alone and significantly higher bioburden than in the powder (Group 3) and bead (Group 4) groups ($p = 0.004$ and $p = 0.014$; Table S3). A similar pattern was observed in screw track samples: although the positive control again had the lowest bioburden, the only significant differences were less bioburden in Group 5 compared to systemic therapy and beads (Groups 2 and 4) ($p = 0.008$, $p = 0.008$; Table S3). No significant differences were observed between groups in bone samples (Table S3).

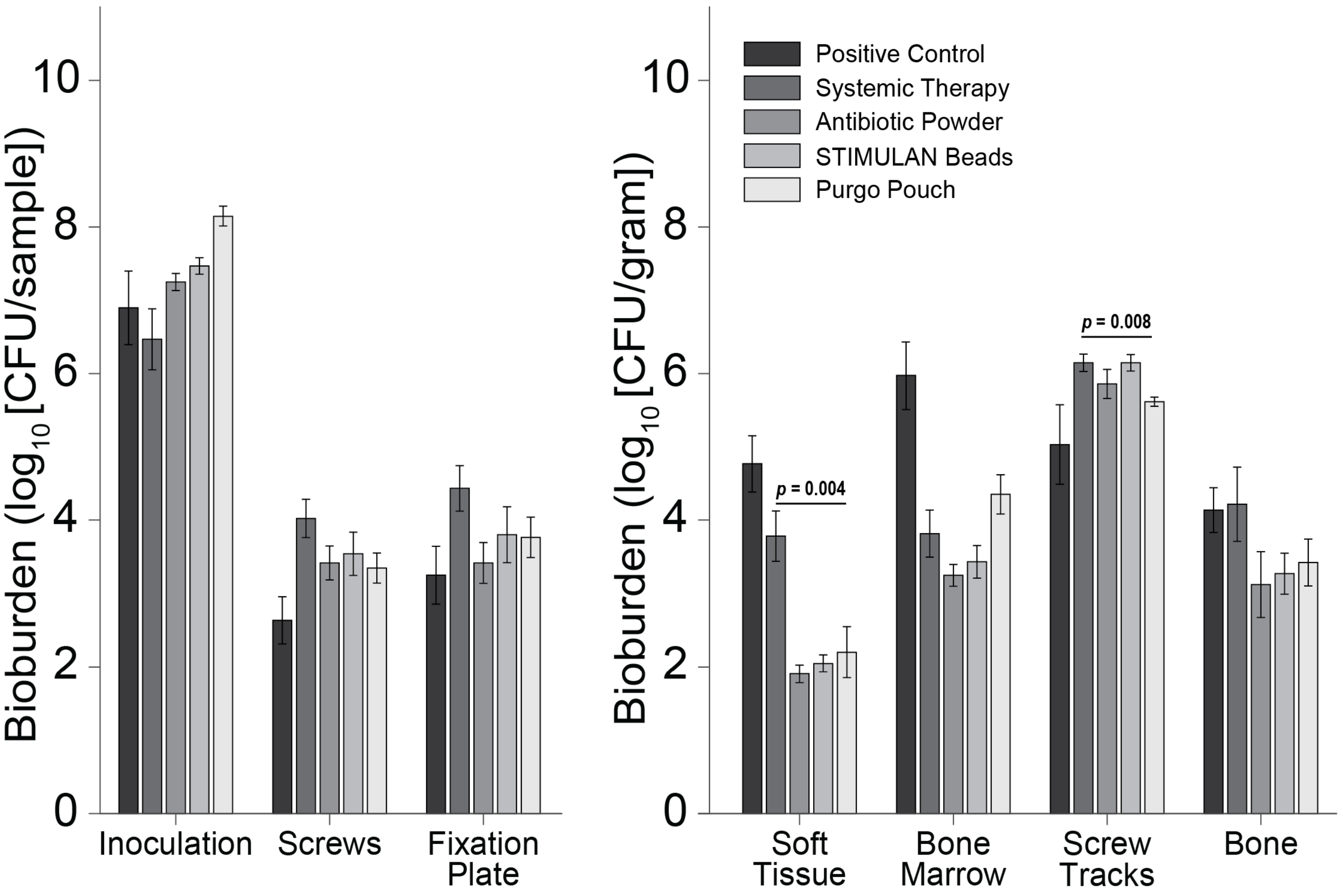

Figure S2. ***Pseudomonas aeruginosa*** **individual sample bioburden**. The average *P. aeruginosa* bioburden (log_{10} units) recovered, broken down by sample. Hardware samples are reported as the total bioburden collected per sample or set of samples (the four screw samples are aggregated), and tissue samples are normalized by weight. Statistical comparisons between the samples are reported in Table S3. Error bars represent ± standard error.

Table S3: *Pseudomonas aeruginosa* microbiology statistical comparisons adjusted with an FDR procedure. Group 1 = Pos. Ctrl.; Group 2 = Systemic Therapy; Group 3 = Antibiotic Powders; Group 4 = Antibiotic Beads; Group 5 = Pouch. Group means were compared using two-sample *t*-tests with unequal variances (Welch *t*-tests). *p*-values were adjusted using a Benjamini-Krieger-Yekutieli two-stage step-up false discovery rate (FDR) procedure to limit the false-positive findings (Type I errors). Statistically significant comparisons ($p < 0.05$) are bold.

**Inoculation**		**Screws**		**Fixation Plate**		**Soft Tissue**		**Bone Marrow**		**Screw Tracks**		**Bone**	
**Group Comparison**	**Adjusted *p*-value**	**Group Compari-son**	**Adjusted *p*-value**	**Group Compari-son**	**Ad-justed *p*-value**	**Group Compari-son**	**Ad-justed *p*-value**	**Group Compari-son**	**Ad-justed *p*-value**	**Group Compari-son**	**Ad-justed *p*-value**	**Group Compari-son**	**Ad-justed *p*-value**
1 vs. 5	0.079	1 vs. 5	0.180	1 vs. 5	0.563	1 vs. 5	**0.000**	1 vs. 5	**0.013**	1 vs. 5	0.311	1 vs. 5	0.279
1 vs. 4	0.288	1 vs. 4	0.180	1 vs. 4	0.563	1 vs. 4	**0.001**	1 vs. 4	**0.004**	1 vs. 4	0.204	1 vs. 4	0.279
1 vs. 3	0.385	1 vs. 3	0.180	1 vs. 3	0.861	1 vs. 3	**0.000**	1 vs. 3	**0.004**	1 vs. 3	0.284	1 vs. 3	0.279
1 vs. 2	0.385	1 vs. 2	0.074	1 vs. 2	0.205	1 vs. 2	0.050	1 vs. 2	**0.004**	1 vs. 2	0.204	1 vs. 2	0.940
2 vs. 5	**0.010**	2 vs. 5	0.180	2 vs. 5	0.455	2 vs. 5	**0.004**	2 vs. 5	0.116	2 vs. 5	**0.008**	2 vs. 5	0.368
2 vs. 4	0.079	2 vs. 4	0.362	2 vs. 4	0.563	2 vs. 4	**0.001**	2 vs. 4	0.161	2 vs. 4	0.836	2 vs. 4	0.279
2 vs. 3	0.131	2 vs. 3	0.180	2 vs. 3	0.205	2 vs. 3	**0.001**	2 vs. 3	0.083	2 vs. 3	0.284	2 vs. 3	0.279
3 vs. 5	**0.000**	3 vs. 5	0.863	3 vs. 5	0.563	3 vs. 5	0.208	3 vs. 5	**0.004**	3 vs. 5	0.287	3 vs. 5	0.890
3 vs. 4	0.207	3 vs. 4	0.863	3 vs. 4	0.563	3 vs. 4	0.208	3 vs. 4	0.211	3 vs. 4	0.284	3 vs. 4	0.912
4 vs. 5	**0.007**	4 vs. 5	0.781	4 vs. 5	0.986	4 vs. 5	0.290	4 vs. 5	**0.014**	4 vs. 5	**0.008**	4 vs. 5	0.912

Table S4: ***Pseudomonas aeruginosa*** **unadjusted microbiology statistical comparisons**. Group 1 = Pos. Ctrl.; Group 2 = Systemic Therapy; Group 3 = Antibiotic Powders; Group 4 = Antibiotic Beads; Group 5 = Pouch. Group means were compared using two-sample *t*-tests with unequal variances (Welch *t*-tests). Statistically significant comparisons ($p < 0.05$) are bold.

**Inoculation**		**Screws**		**Fixation Plate**		**Soft Tissue**		**Bone Marrow**		**Screw Tracks**		**Bone**	
**Group Comparison**	**Adjusted *p*-value**	**Group Comparison**	**Adjusted *p*-value**	**Group Comparison**	**Adjusted *p*-value**	**Group Comparison**	**Adjusted *p*-value**	**Group Comparison**	**Adjusted *p*-value**	**Group Comparison**	**Adjusted *p*-value**	**Group Comparison**	**Adjusted *p*-value**
1 vs. 5	0.054	1 vs. 5	0.095	1 vs. 5	0.310	1 vs. 5	**0.000**	1 vs. 5	**0.016**	1 vs. 5	0.333	1 vs. 5	0.133
1 vs. 4	0.313	1 vs. 4	0.061	1 vs. 4	0.334	1 vs. 4	**0.001**	1 vs. 4	**0.002**	1 vs. 4	0.096	1 vs. 4	0.060
1 vs. 3	0.522	1 vs. 3	0.078	1 vs. 3	0.738	1 vs. 3	**0.000**	1 vs. 3	**0.001**	1 vs. 3	0.200	1 vs. 3	0.087
1 vs. 2	0.524	1 vs. 2	**0.007**	1 vs. 2	**0.039**	1 vs. 2	0.083	1 vs. 2	**0.004**	1 vs. 2	0.097	1 vs. 2	0.895
2 vs. 5	**0.004**	2 vs. 5	0.061	2 vs. 5	0.130	2 vs. 5	**0.006**	2 vs. 5	0.221	2 vs. 5	**0.002**	2 vs. 5	0.210
2 vs. 4	**0.049**	2 vs. 4	0.241	2 vs. 4	0.220	2 vs. 4	**0.001**	2 vs. 4	0.344	2 vs. 4	0.995	2 vs. 4	0.130
2 vs. 3	0.107	2 vs. 3	0.103	2 vs. 3	**0.029**	2 vs. 3	**0.001**	2 vs. 3	0.139	2 vs. 3	0.237	2 vs. 3	0.128
3 vs. 5	**0.000**	3 vs. 5	0.822	3 vs. 5	0.388	3 vs. 5	0.446	3 vs. 5	**0.004**	3 vs. 5	0.273	3 vs. 5	0.593
3 vs. 4	0.197	3 vs. 4	0.743	3 vs. 4	0.429	3 vs. 4	0.412	3 vs. 4	0.503	3 vs. 4	0.231	3 vs. 4	0.782
4 vs. 5	**0.002**	4 vs. 5	0.595	4 vs. 5	0.939	4 vs. 5	0.691	4 vs. 5	**0.020**	4 vs. 5	**0.002**	4 vs. 5	0.726

A.1.3 Combined Staphylococcus aureus and Pseudomonas aeruginosa microbiology

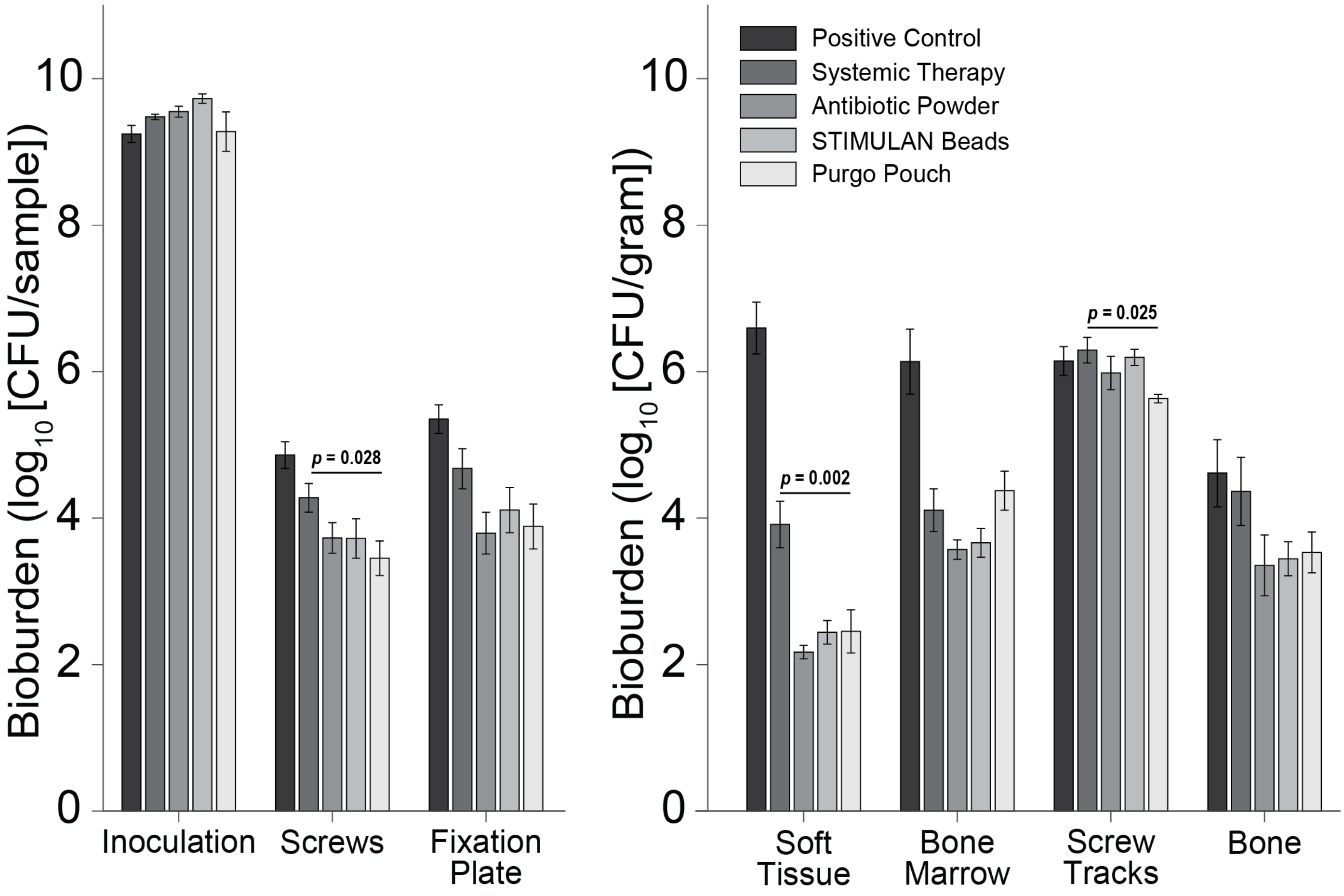

Figure S3: Combined *Staphylococcus aureus* and *Pseudomonas aeruginosa* sample bioburden. For each sample, the reported value is the average bioburden (log_{10} units) calculated from the combined recovery of *S. aureus* and *P. aeruginosa*. Hardware samples are reported as the total bioburden collected per sample or set of samples (the four screw samples are aggregated), and tissue samples are normalized by weight. Statistical comparisons between the samples are reported in Table S5 below. Error bars represent ± standard error.

Inoculation		Screws		Fixation Plate		Soft Tissue		Bone Marrow		Screw Tracks		Bone	
**Group Comparison**	**Adjusted *p*-value**	**Group Comparison**	**Adjusted *p*-value**	**Group Comparison**	**Adjusted *p*-value**	**Group Comparison**	**Adjusted *p*-value**	**Group Comparison**	**Adjusted *p*-value**	**Group Comparison**	**Adjusted *p*-value**	**Group Comparison**	**Adjusted *p*-value**
1 vs. 5	0.769	1 vs. 5	**0.006**	1 vs. 5	**0.007**	1 vs. 5	**0.000**	1 vs. 5	**0.011**	1 vs. 5	0.132	1 vs. 5	0.268
1 vs. 4	**0.029**	1 vs. 4	**0.011**	1 vs. 4	**0.015**	1 vs. 4	**0.000**	1 vs. 4	**0.005**	1 vs. 4	0.701	1 vs. 4	0.268
1 vs. 3	0.154	1 vs. 3	**0.006**	1 vs. 3	**0.007**	1 vs. 3	**0.000**	1 vs. 3	**0.005**	1 vs. 3	0.596	1 vs. 3	0.268
1 vs. 2	0.173	1 vs. 2	0.064	1 vs. 2	0.094	1 vs. 2	**0.000**	1 vs. 2	**0.007**	1 vs. 2	0.596	1 vs. 2	0.897
2 vs. 5	0.451	2 vs. 5	**0.028**	2 vs. 5	0.094	2 vs. 5	**0.002**	2 vs. 5	0.298	2 vs. 5	**0.025**	2 vs. 5	0.268
2 vs. 4	**0.029**	2 vs. 4	0.108	2 vs. 4	0.203	2 vs. 4	**0.001**	2 vs. 4	0.150	2 vs. 4	0.596	2 vs. 4	0.268
2 vs. 3	0.439	2 vs. 3	0.080	2 vs. 3	0.079	2 vs. 3	**0.001**	2 vs. 3	0.092	2 vs. 3	0.496	2 vs. 3	0.268
3 vs. 5	0.432	3 vs. 5	0.312	3 vs. 5	0.609	3 vs. 5	0.136	3 vs. 5	**0.023**	3 vs. 5	0.368	3 vs. 5	0.897
3 vs. 4	0.173	3 vs. 4	0.621	3 vs. 4	0.427	3 vs. 4	0.069	3 vs. 4	0.369	3 vs. 4	0.588	3 vs. 4	0.897
4 vs. 5	0.203	4 vs. 5	0.324	4 vs. 5	0.503	4 vs. 5	0.306	4 vs. 5	**0.046**	4 vs. 5	**0.008**	4 vs. 5	0.897

Table S5: Combined *Staphylococcus aureus and Pseudomonas aeruginosa* microbiology statistical comparisons adjusted with an FDR procedure. Group 1 = Pos. Ctrl.; Group 2 = Systemic Therapy; Group 3 = Antibiotic Powders; Group 4 = Antibiotic Beads; Group 5 = Pouch. Group means were compared using two-sample *t*-tests with unequal variances (Welch *t*-tests). *p*-values were adjusted using a Benjamini-Krieger-Yekutieli two-stage step-up false discovery rate (FDR) procedure to limit the false-positive findings (Type I errors). Statistically significant comparisons ($p < 0.05$) are bold.

Table S6: Combined *Staphylococcus aureus and Pseudomonas aeruginosa* unadjusted microbiology statistical comparisons. Group 1 = Pos. Ctrl.; Group 2 = Systemic Therapy; Group 3 = Antibiotic Powders; Group 4 = Antibiotic Beads; Group 5 = Pouch. Group means were compared using two-sample *t*-tests with unequal variances (Welch *t*-tests). Statistically significant comparisons ($p < 0.05$) are bold.

Inoculation		Screws		Fixation Plate		Soft Tissue		Bone Marrow		Screw Tracks		Bone	
**Group Comparison**	**Adjusted *p*-value**	**Group Comparison**	**Adjusted *p*-value**	**Group Comparison**	**Adjusted *p*-value**	**Group Comparison**	**Adjusted *p*-value**	**Group Comparison**	**Adjusted *p*-value**	**Group Comparison**	**Adjusted *p*-value**	**Group Comparison**	**Adjusted *p*-value**
1 vs. 5	0.916	1 vs. 5	**0.001**	1 vs. 5	**0.002**	1 vs. 5	**0.000**	1 vs. 5	**0.008**	1 vs. 5	**0.047**	1 vs. 5	0.078
1 vs. 4	**0.007**	1 vs. 4	**0.005**	1 vs. 4	**0.006**	1 vs. 4	**0.000**	1 vs. 4	**0.001**	1 vs. 4	0.835	1 vs. 4	0.056
1 vs. 3	0.055	1 vs. 3	**0.002**	1 vs. 3	**0.001**	1 vs. 3	**0.000**	1 vs. 3	**0.002**	1 vs. 3	0.595	1 vs. 3	0.067
1 vs. 2	0.103	1 vs. 2	0.051	1 vs. 2	0.068	1 vs. 2	**0.000**	1 vs. 2	**0.004**	1 vs. 2	0.583	1 vs. 2	0.708
2 vs. 5	0.483	2 vs. 5	**0.018**	2 vs. 5	0.077	2 vs. 5	**0.005**	2 vs. 5	0.511	2 vs. 5	**0.006**	2 vs. 5	0.153
2 vs. 4	**0.007**	2 vs. 4	0.120	2 vs. 4	0.193	2 vs. 4	**0.002**	2 vs. 4	0.228	2 vs. 4	0.638	2 vs. 4	0.107
2 vs. 3	0.418	2 vs. 3	0.076	2 vs. 3	**0.043**	2 vs. 3	**0.001**	2 vs. 3	0.123	2 vs. 3	0.295	2 vs. 3	0.128
3 vs. 5	0.360	3 vs. 5	0.396	3 vs. 5	0.829	3 vs. 5	0.387	3 vs. 5	**0.022**	3 vs. 5	0.175	3 vs. 5	0.728
3 vs. 4	0.094	3 vs. 4	0.985	3 vs. 4	0.465	3 vs. 4	0.174	3 vs. 4	0.702	3 vs. 4	0.420	3 vs. 4	0.854
4 vs. 5	0.145	4 vs. 5	0.463	4 vs. 5	0.616	4 vs. 5	0.970	4 vs. 5	0.052	4 vs. 5	**0.001**	4 vs. 5	0.812

2.1 Individual histological sample comparisons

Table S7: Histological statistical comparisons adjusted with an FDR procedure. The woven bone samples were compared with Welch's *t*-tests (unequal variance), and the osteoclast and bone volume samples were compared using mixed-effect linear regression models. Group -1 = Time = 0; Group 0 = Negative Control; Group 1= Positive Control; Group 2 = Systemic Therapy; Group 3 = Antibiotic Powders; Group 4 = Antibiotic Beads; Group 5 = Pouch. *p*-values were adjusted using a Benjamini-Krieger-Yekutieli two-stage step-up false discovery rate (FDR) procedure to limit the false-positive findings (Type I errors). Statistically significant comparisons ($p < 0.05$) are bold.

**Osteoclast Count**		**Bone Volume (μCT)**		**Woven Bone (SEM)**	
**Group Comparison**	**Adjusted *p*-value**	**Group Comparison**	**Adjusted *p*-value**	**Group Comparison**	**Adjusted *p*-value**
-1 vs. 0	0.567	-1 vs. 0	0.361	-1 vs. 0	**0.008**
-1 vs. 1	0.287	-1 vs. 1	0.404	-1 vs. 1	0.339
-1 vs. 2	**0.018**	-1 vs. 2	**0.000**	-1 vs. 2	0.161
-1 vs. 3	0.202	-1 vs. 3	**0.001**	-1 vs. 3	**0.037**
-1 vs. 4	**0.005**	-1 vs. 4	**0.000**	-1 vs. 4	0.082
-1 vs. 5	0.324	-1 vs. 5	0.198	-1 vs. 5	0.161
0 vs. 1	0.202	0 vs. 1	0.252	0 vs. 1	**0.008**
0 vs. 2	**0.000**	0 vs. 2	**0.000**	0 vs. 2	0.208
0 vs. 3	0.079	0 vs. 3	**0.000**	0 vs. 3	0.678
0 vs. 4	**0.000**	0 vs. 4	**0.000**	0 vs. 4	**0.011**
0 vs. 5	0.284	0 vs. 5	0.252	0 vs. 5	0.059
1 vs. 2	**0.020**	1 vs. 2	**0.000**	1 vs. 2	0.167
1 vs. 3	0.385	1 vs. 3	**0.000**	1 vs. 3	**0.037**
1 vs. 4	**0.003**	1 vs. 4	**0.000**	1 vs. 4	0.123
1 vs. 5	0.525	1 vs. 5	0.079	1 vs. 5	0.172
2 vs. 3	0.064	2 vs. 3	0.361	2 vs. 3	0.305
2 vs. 4	0.284	2 vs. 4	0.404	2 vs. 4	0.317
2 vs. 5	**0.019**	2 vs. 5	**0.000**	2 vs. 5	0.486
3 vs. 4	**0.011**	3 vs. 4	0.403	3 vs. 4	0.065
3 vs. 5	0.324	3 vs. 5	**0.001**	3 vs. 5	0.123
4 vs. 5	**0.003**	4 vs. 5	**0.000**	4 vs. 5	0.481

Table S8: Histological unadjusted statistical comparisons. The woven bone samples were compared with Welch's *t*-tests (unequal variance), and the osteoclast and bone volume samples were compared using mixed-effect linear regression models. Group -1 = Time = 0; Group 0 = Negative Control; Group 1= Positive Control; Group 2 = Systemic Therapy; Group 3 = Antibiotic Powders; Group 4 = Antibiotic Beads; Group 5 = Pouch. Statistically significant comparisons ($p < 0.05$) are bold.

**Osteoclast Count**		**Bone Volume (µCT)**		**Woven Bone (SEM)**	
**Group Comparison**	***p*-value**	**Group Comparison**	***p*-value**	**Group Comparison**	***p*-value**
-1 vs. 0	0.945	-1 vs. 0	0.651	-1 vs. 0	**0.001**
-1 vs. 1	0.365	-1 vs. 1	0.898	-1 vs. 1	0.363
-1 vs. 2	**0.010**	-1 vs. 2	**0.000**	-1 vs. 2	0.110
-1 vs. 3	0.199	-1 vs. 3	**0.001**	-1 vs. 3	**0.010**
-1 vs. 4	**0.002**	-1 vs. 4	**0.000**	-1 vs. 4	**0.039**
-1 vs. 5	0.463	-1 vs. 5	0.293	-1 vs. 5	0.115
0 vs. 1	0.208	0 vs. 1	0.427	0 vs. 1	**0.001**
0 vs. 2	**0.000**	0 vs. 2	**0.000**	0 vs. 2	0.186
0 vs. 3	0.069	0 vs. 3	**0.000**	0 vs. 3	0.848
0 vs. 4	**0.000**	0 vs. 4	**0.000**	0 vs. 4	**0.002**
0 vs. 5	0.328	0 vs. 5	0.424	0 vs. 5	**0.021**
1 vs. 2	**0.014**	1 vs. 2	**0.000**	1 vs. 2	0.129
1 vs. 3	0.581	1 vs. 3	**0.000**	1 vs. 3	**0.011**
1 vs. 4	**0.001**	1 vs. 4	**0.000**	1 vs. 4	0.066
1 vs. 5	0.833	1 vs. 5	0.108	1 vs. 5	0.143
2 vs. 3	0.051	2 vs. 3	0.687	2 vs. 3	0.290
2 vs. 4	0.338	2 vs. 4	0.870	2 vs. 4	0.321
2 vs. 5	**0.012**	2 vs. 5	**0.000**	2 vs. 5	0.578
3 vs. 4	**0.005**	3 vs. 4	0.810	3 vs. 4	**0.027**
3 vs. 5	0.463	3 vs. 5	**0.001**	3 vs. 5	0.073
4 vs. 5	**0.001**	4 vs. 5	**0.000**	4 vs. 5	0.544

3.1 Effect size analysis

Table S9: Effect size for *S. aureus* hardware reduction scores. Group 1= Positive Control; Group 2 = Systemic Therapy; Group 3 = Antibiotic Powders; Group 4 = Antibiotic Beads; Group 5 = Pouch. comparisons performed using Welch's two-sample *t*-test. Cohen's d was calculated using the pooled standard deviations. Effect size benchmarks: negligible d < 0.2, small d = 0.2, medium d = 0.5, large d ≥ 0.8. Group A refers to the first group in the group comparison column; Group B refers to the second group in the group comparison column.

Group Comparison	Group A Mean ± SD (log_{10} CFUs)	Group B Mean ± SD (log_{10} CFUs)	Mean Difference	*p*-value	Cohen's d (95% CI)	Interpretation
1 vs. 5	3.39 ± 0.68	5.65 ± 1.57	-2.26	**0.011**	1.76 (0.49, 3.03)	Large
1 vs. 4	3.39 ± 0.68	5.66 ± 0.85	-2.27	**< 0.001**	2.88 (1.32, 4.45)	Large
1 vs. 3	3.39 ± 0.68	5.69 ± 1.44	-2.30	**0.01**	1.93 (0.62, 3.24)	Large
1 vs. 2	3.39 ± 0.68	5.03 ± 1.22	-1.64	**0.013**	1.59 (0.35, 2.82)	Large
2 vs. 5	5.03 ± 1.22	5.65 ± 1.57	-0.62	0.355	0.44 (-0.55, 1.43)	Small
2 vs. 4	5.03 ± 1.22	5.66 ± 0.85	-0.63	0.319	0.60 (-0.41, 1.60)	Medium
2 vs. 3	5.03 ± 1.22	5.69 ± 1.44	-0.66	0.355	0.49 (-0.50, 1.49)	Small
3 vs. 5	5.69 ± 1.44	5.65 ± 1.57	+0.04	0.622	0.03 (-0.95, 1.01)	Negligible
3 vs. 4	5.69 ± 1.44	5.66 ± 0.85	+0.03	0.622	0.03 (-0.96, 1.01)	Negligible
4 vs. 5	5.66 ± 0.85	5.65 ± 1.57	+0.01	0.622	0.01 (-0.97, 0.99)	Negligible

Table S10: Effect size for *P. aeruginosa* hardware reduction scores. Group 1= Positive Control; Group 2 = Systemic Therapy; Group 3 = Antibiotic Powders; Group 4 = Antibiotic Beads; Group 5 = Pouch. comparisons performed using Welch's two-sample *t*-test. Cohen's d was calculated using the pooled standard deviations. Effect size benchmarks: negligible d < 0.2, small d = 0.2, medium d = 0.5, large d ≥ 0.8. Group A refers to the first group in the group comparison column; Group B refers to the second group in the group comparison column.

Group Comparison	Group A Mean ± SD (log_{10} CFU)	Group B Mean ± SD (log_{10} CFU)	Mean Difference	*p*-value	Cohen's d (95% CI)	Interpretation
1 vs. 5	3.05 ± 1.17	3.64 ± 0.55	-0.59	0.219	0.68 (-0.41, 1.78)	Medium
1 vs. 4	3.05 ± 1.17	2.79 ± 0.89	0.26	0.388	0.25 (-0.81, 1.32)	Small
1 vs. 3	3.05 ± 1.17	2.74 ± 0.55	0.31	0.375	0.36 (-0.71, 1.42)	Small
1 vs. 2	3.05 ± 1.17	1.33 ± 1.03	1.72	**0.018**	1.58 (0.34, 2.81)	Large
2 vs. 5	1.33 ± 1.03	3.64 ± 0.55	-2.31	**< 0.001**	2.79 (1.37, 4.22)	Large
2 vs. 4	1.33 ± 1.03	2.79 ± 0.89	-1.46	**0.012**	1.52 (0.39, 2.65)	Large
2 vs. 3	1.33 ± 1.03	2.74 ± 0.55	-1.41	**0.011**	1.71 (0.54, 2.88)	Large
3 vs. 5	2.74 ± 0.55	3.64 ± 0.55	-0.90	**0.011**	1.63 (0.48, 2.79)	Large
3 vs. 4	2.74 ± 0.55	2.79 ± 0.89	-0.05	0.468	0.07 (-0.91, 1.05)	Negligible
4 vs. 5	2.79 ± 0.89	3.64 ± 0.55	-0.85	**0.037**	1.14 (0.07, 2.21)	Large

Table S11: Effect size for *S. aureus* tissue bioburden. Group 1= Positive Control; Group 2 = Systemic Therapy; Group 3 = Antibiotic Powders; Group 4 = Antibiotic Beads; Group 5 = Pouch. comparisons performed using Welch's two-sample *t*-test. Cohen's d was calculated using the pooled standard deviations. Effect size benchmarks: negligible d < 0.2, small d = 0.2, medium d = 0.5, large d ≥ 0.8. Group A refers to the first group in the group comparison column; Group B refers to the second group in the group comparison column.

Group Comparison	Group A Mean ± SD (log_{10} CFU)	Group B Mean ± SD (log_{10} CFU)	Mean Difference	*p*-value	Cohen's d (95% CI)	Interpretation
1 vs. 5	7.22 ± 0.62	3.60 ± 0.91	3.62	**< 0.001**	4.51 (2.42, 6.60)	Large
1 vs. 4	7.22 ± 0.62	4.69 ± 0.84	2.53	**< 0.001**	3.36 (1.65, 5.07)	Large
1 vs. 3	7.22 ± 0.62	4.80 ± 1.20	2.42	**0.001**	2.43 (0.99, 3.87)	Large
1 vs. 2	7.22 ± 0.62	5.40 ± 0.74	1.82	**< 0.001**	2.63 (1.14, 4.12)	Large
2 vs. 5	5.40 ± 0.74	3.60 ± 0.91	1.80	**0.001**	2.16 (0.90, 3.43)	Large
2 vs. 4	5.40 ± 0.74	4.69 ± 0.84	0.71	0.05	0.90 (-0.14, 1.93)	Large
2 vs. 3	5.40 ± 0.74	4.80 ± 1.20	0.60	0.116	0.61 (-0.40, 1.61)	Medium
3 vs. 5	4.80 ± 1.20	3.60 ± 0.91	1.20	**0.026**	1.12 (0.06, 2.19)	Large
3 vs. 4	4.80 ± 1.20	4.69 ± 0.84	0.11	0.354	0.10 (-0.88, 1.08)	Negligible
4 vs. 5	4.69 ± 0.84	3.60 ± 0.91	1.09	**0.018**	1.25 (0.16, 2.33)	Large

Table S12: Effect size for *P. aeruginosa* tissue bioburden. Group 1= Positive Control; Group 2 = Systemic Therapy; Group 3 = Antibiotic Powders; Group 4 = Antibiotic Beads; Group 5 = Pouch. comparisons performed using Welch's two-sample *t*-test. Cohen's d was calculated using the pooled standard deviations. Effect size benchmarks: negligible d < 0.2, small d = 0.2, medium d = 0.5, large d ≥ 0.8. Group A refers to the first group in the group comparison column; Group B refers to the second group in the group comparison column.

Group Comparison	Group A Mean ± SD (log_{10} CFU)	Group B Mean ± SD (log_{10} CFU)	Mean Difference	*p*-value	Cohen's d (95% CI)	Interpretation
1 vs. 5	6.06 ± 0.44	5.71 ± 0.17	0.35	0.309	1.11 (-0.04, 2.26)	Large
1 vs. 4	6.06 ± 0.44	6.03 ± 0.23	0.03	0.749	0.08 (-0.98, 1.14)	Negligible
1 vs. 3	6.06 ± 0.44	5.77 ± 0.58	0.29	0.437	0.55 (-0.53, 1.63)	Medium
1 vs. 2	6.06 ± 0.44	6.13 ± 0.30	-0.07	0.744	0.21 (-0.86, 1.27)	Small
2 vs. 5	6.13 ± 0.30	5.71 ± 0.17	0.42	**0.038**	1.71 (0.55, 2.88)	Large
2 vs. 4	6.13 ± 0.30	6.03 ± 0.23	0.10	0.553	0.38 (-0.61, 1.37)	Small
2 vs. 3	6.13 ± 0.30	5.77 ± 0.58	0.36	0.309	0.78 (-0.24, 1.81)	Medium
3 vs. 5	5.77 ± 0.58	5.71 ± 0.17	0.06	0.744	0.13 (-0.85, 1.11)	Negligible
3 vs. 4	5.77 ± 0.58	6.03 ± 0.23	-0.26	0.437	0.59 (-0.42, 1.59)	Medium
4 vs. 5	6.03 ± 0.23	5.71 ± 0.17	0.32	**0.038**	1.54 (0.41, 2.67)	Large
